# The Effects of Sequence Structure on the Mechanical Properties of Siloxane-Containing Polyimides: Insights from Molecular Dynamics Simulations

**DOI:** 10.3390/ijms27167248

**Published:** 2026-08-14

**Authors:** Lixin Liu, Song Mo, Fan Jia, Yi Liu, Lei Zhai, Lin Fan

**Affiliations:** 1 Key Laboratory of Science and Technology on High-Tech Polymer Materials, Institute of Chemistry, Chinese Academy of Sciences, Beijing 100190, China; 2 School of Chemical Sciences, University of Chinese Academy of Sciences, Beijing 100049, China

**Keywords:** siloxane-containing polyimide, sequence structure, molecular dynamics simulations, mechanical properties

## Abstract

In order to provide a theoretical framework for the synergistic optimization of the “rigid backbone-flexible network” in the molecular design of polyimides with high Young’s modulus, high toughness, and excellent creep resistance for wearable electronics applications, the effects of sequence structure on the mechanical properties of siloxane-containing polyimides were investigated by molecular dynamics simulations. A series of poly(siloxane-imide) block copolymer models with distinct sequence structures were constructed via molecular dynamics (MD) simulations based on 4,4′-(hexafluoroisopropylidene)diphthalic anhydride (6FDA) and 2,2′-bis(trifluoromethyl)benzidine (TFDB) as hard segment A, and 6FDA and 1,3-bis(3-aminopropyl)tetramethyldisiloxane (SiDA) as soft segment B. The results indicate that extending hard segment length enhances Young’s modulus and suppresses creep because of the enhancement of chain rigidity and formation of stable physical aggregates. Appropriately extending soft segment sequence length can improve the failure strain through rapid conformational adjustment, while excessively long soft segments lead to stress concentration, thereby reducing the failure strain. The (A_5_B_5_)_2_ model structure exhibits superior comprehensive performance among all systems, with a relatively high Young’s modulus, failure strain, and creep recovery rate. This is attributed to the synergistic balance between the rigidity of the hard segment and the mobility of the soft segment.

## 1. Introduction

In recent years, flexible and stretchable electronic devices have successfully overcome the rigid physical limitations of traditional silicon-based electronic devices. As wearable electronic sensors, they demonstrate enormous application potential in cutting-edge fields such as real-time medical monitoring, bionic robot perception, and intelligent interactive interfaces [1,2,3,4,5,6,7,8]. As the mechanical support structure for flexible wearable electronic devices, substrate materials need to possess excellent stretchability and long-term reliability under sustained stress, which poses a significant challenge to existing material systems. High-elasticity materials, such as polydimethylsiloxane (PDMS) [9,10], Ecoflex silicone rubber [11,12], and thermoplastic polyurethane (TPU) [13,14], are commonly used to construct stretchable substrates due to their excellent stretchability. However, these materials exhibit low elastic modulus and insufficient creep resistance, leading to plastic deformation under cyclic loading, which further results in interfacial delamination and degradation of electrical performance in devices [15,16]. In contrast, polyimide (PI) has become a dominant substrate material in the field of flexible printed circuits (FPCs) [17,18], owing to its outstanding comprehensive properties, including high strength and toughness, excellent heat resistance, and low dielectric constant. Nevertheless, the inherent limitation of traditional PI films in terms of stretchability significantly restricts their application expansion in the field of wearable electronics.

As an effort to develop stretchable polyimide films, many researchers have been working on incorporating flexible polysiloxane segments into the main chain of polyimide [19,20]. Ghosh et al. [21] synthesized siloxane-containing polyimide copolymers by incorporating aminopropyl-terminated polydimethylsiloxane (APPS) segments into polyimide derived from 4,4′-(4,4′-isopropylidenediphenoxy)bis(phthalic anhydride) (BPADA) and 4,4′-oxydianiline (ODA). They found that both the toughness and elongation at break of the copolyimide films significantly increased with the increasing of siloxane content. Compared with the BPADA/ODA homopolymer, the R80 copolymer film with an APPS-to-ODA ratio of 4:1 exhibited a remarkable increase in toughness from 215 kJ/m^3^ to 2300 kJ/m^3^, along with a rise in elongation at break from 5% to 333%. This enhancement in film toughness was explained by the higher free volume and mobile soft segments in the siloxane-containing copolymer system, which enable effective energy dissipation and thus endow the film with excellent stretchability. However, due to the disorder in sequence distribution of the rigid and flexible segments in the random copolymer, it is difficult to precisely regulate the overall properties of the material.

Pei et al. [22] incorporated the polysiloxane segment APPS into the polyimide based on 3,3′,4,4′-thiodiphenyl dianhydride (TDPA)/4,4′-oxydianiline (ODA), and successfully synthesized a series of block copolymers with controllable lengths of polyimide hard segments and polysiloxane soft segments. It was found that as the length of the polyimide hard segments increased, the storage modulus of these block copolymers also increased and was significantly higher than that of the corresponding random copolymers. It was also noticed that the Young’s modulus of all block copolymers was obviously lower than that of the corresponding random copolymers. However, all block copolymers exhibited higher elastic recovery rates compared to the random copolymers under the same strain conditions, and these rates increased further with block length. This phenomenon was attributed to the extension of the polysiloxane soft segment in the block copolymer, whose mobility increases with segment length, thereby significantly improving elastic recovery capacity while reducing Young’s modulus. Although many studies have made considerable progress in enhancing the stretchability of polyimide materials by introducing polysiloxane segments, the problem of simultaneous decrease in Young’s modulus still needs to be addressed. In addition, traditional experimental methods are largely confined to investigating how the chemical structure of multicomponent copolymer systems affects macroscopic properties, while remaining insufficient in efficiently regulating the hard/soft segment ratio and elucidating the structure–property relationship between sequence distribution and macroscopic properties at the molecular scale.

As a mature computer-aided technology, molecular simulation has demonstrated indispensable advantages in the field of polymer material design and property prediction after years of development [23,24,25,26]. Through simulating and calculating specific polymer structures, molecular simulation can not only predict the macroscopic properties of materials but also deeply explore the influence mechanism of the intrinsic microstructure of polymers on macroscopic properties, thereby providing a theoretical basis for experimental laws [27,28,29,30,31]. Xiang and his coworkers [32] reported their research on the structure–property relationship between the mechanical properties and glass transition temperature (T_g_) of polylactic acid (PLA)-polyethylene glycol (PEG) block copolymers via molecular dynamics (MD) simulations. The good agreement between the simulation results and experimental data verified the reliability of this method. Ji et al. [33] constructed a molecular model of diethyl–dimethyl–diphenyl ternary silicone copolymer (EMP-SR) via MD simulations by introducing diphenylsiloxane and dimethylsiloxane segments into poly(diethylsiloxane) (PDES), and predicted the influence of the content ratio between diethylsiloxane and dimethylsiloxane segments on the T_g_. This prediction successfully guided the synthesis of a high-performance elastomer with ultra-low temperature resistance. Jiang et al. [34] systematically constructed molecular models of poly(ether-b-amide) and poly(ester-b-amide) copolymers using all-atom MD simulations. These copolymers feature long-chain polyamide (PA 1212) as the hard segments and various polyethers or polyesters as the soft segments. The simulation results revealed that the structure of soft segments and their content in the copolymer directly determine the strain hardening degree and tensile strength of the material by regulating the strain-induced crystallization behavior. Among them, poly(tetramethylene glycol) (PTMG) and polycaprolactone (PCL) are more prone to strain hardening and exhibit excellent tensile strength. During deformation, the hydrogen bonding interactions decreased as the strain increased, which suggested that the hydrogen bond density of the polyamide hard segments played a dominant role. This modeling method reveals the molecular-level mechanism of strain hardening in polyamide block copolymers and provides a theoretical basis for the precise design of thermoplastic polyamide elastomers. Avaz et al. [35] constructed molecular models of segmented polyurethane–urea block copolymers, in which poly(ethylene oxide) (PEO) serves as the soft segments (SS) and the urea linkages/urea groups formed by bis(4-isocyanatocyclohexyl)methane (HMDI) (a diisocyanate) and 2-methyl-1,5-diaminopentane (MDAP) (a diamine chain extender) act as the hard segments (HS), using a multi-scale simulation approach combining MD and dissipative particle dynamics (DPD). The influence of SS chain length on the copolymer’s morphology and structure–property relationships was investigated. The results revealed that the critical chain length of SS plays a key role in the structure–property relationships of these copolymers. Below the critical SS length, intermolecular interactions between HS and SS dominate the final morphological development, forming a typical phase-blended structure. Above the critical SS length, interactions within SS dominate the morphological development of both copolymers, leading to a phase-separated structure.

These studies have demonstrated that MD simulations can effectively reveal the intrinsic correlations among the sequence structure, microscopic aggregation state, and macroscopic properties of block copolymers, providing a reliable theoretical foundation for the molecular design of high-performance polymer materials. However, molecular simulation studies focusing on siloxane-modified polyimide block copolymer systems are still very limited. According to the previous experimental studies on siloxane-containing polyimide by our research group, it has been found that the mechanical properties and creep resistance of poly(siloxane-imide) are determined by multiple factors. Therefore, it is necessary to elucidate the underlying molecular-level mechanisms to provide theoretical guidance for the molecular design of siloxane-containing polyimides with an excellent balance of multiple properties for wearable electronics applications.

In order to provide a theoretical framework for the synergistic optimization of the “rigid backbone-flexible network” in the molecular design of polyimides with a combination of high Young’s modulus, high toughness, and excellent creep resistance, the effects of sequence structure on the mechanical properties of siloxane-containing polyimides were investigated by molecular dynamics simulations. A series of siloxane-containing polyimides were designed based on fluorinated aromatic dianhydride 4,4′-(hexafluoroisopropylidene) diphthalic anhydride (6FDA), fluorinated aromatic diamine 2,2′-bis(trifluoromethyl)-4,4′-diaminobiphenyl (TFDB), and siloxane-containing diamine bis(4-aminophenoxy)dimethylsilane (SiDA). Two kinds of homopolymer polyimide molecular models 6FDA/TFDB (A_20_) and 6FDA/SiDA (B_20_), along with four kinds of block copolymer polyimide molecular models with distinct sequence structures composed of hard segment A (6FDA/TFDB) and soft segment B (6FDA/SiDA), defined as (A_2_B_2_)_5_, (A_5_B_5_)_2_, A_5_B_10_A_5_, and B_5_A_10_B_5_, were constructed. The effects of sequence distribution parameters, including block length and block arrangement pattern, on the microscopic chain conformation and aggregation structure, as well as macroscopic tensile and creep properties of the copolymer systems, were systematically investigated.

## 2. Results and Discussion

### 2.1. Model Validation

**Figure 1:**
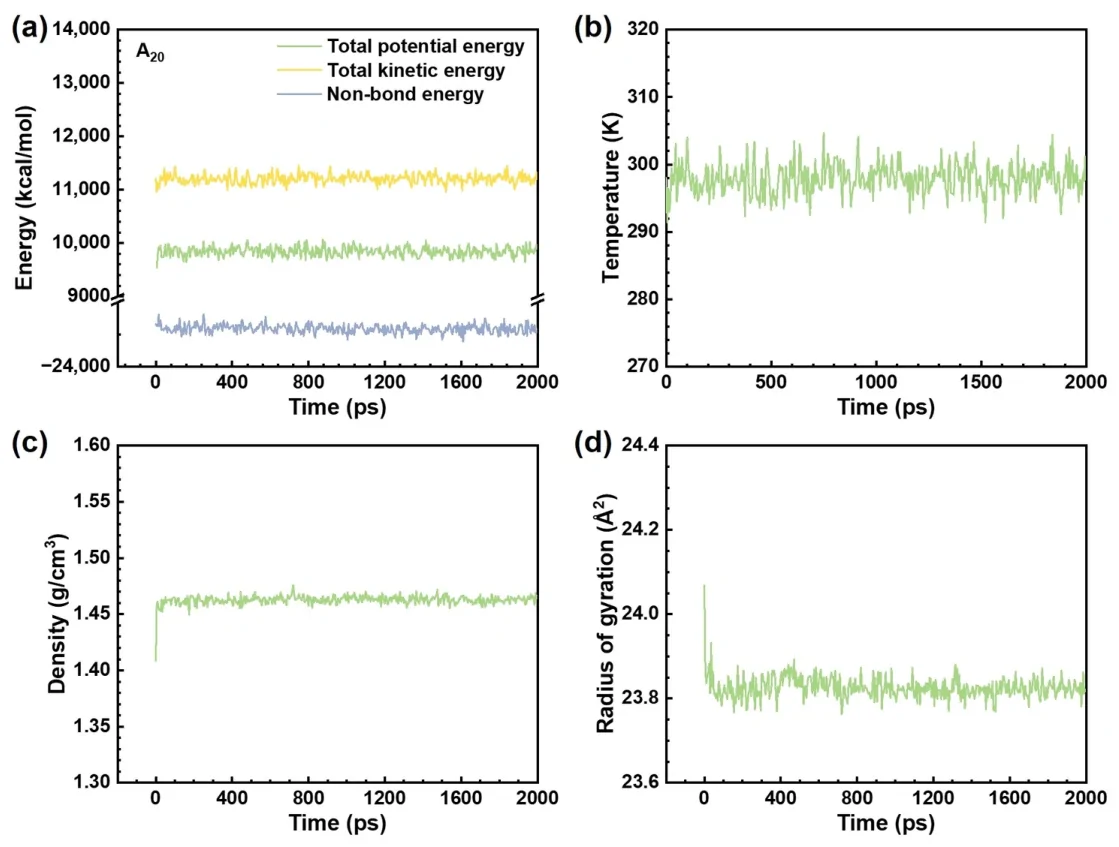
Time evolution of energy (**a**), temperature (**b**), density (**c**), and radius of gyration (**d**) for the A_20_ system during NPT equilibration.

Two kinds of homopolymer polyimide molecular models, 6FDA/TFDB (A_20_) and 6FDA/SiDA (B_20_), along with four kinds of poly(siloxane-imide) block copolymer polyimide molecular models, were constructed using BIOVIA Materials Studio 2019 (version 19.1) software. All systems underwent multi-step high-temperature annealing followed by NPT equilibration at room temperature to obtain fully relaxed amorphous structures. The equilibrium state of each system was comprehensively validated based on the time evolution of energy, temperature, density, and radius of gyration. Taking the A_20_ system as a representative example shown in Figure 1, all key indicators exhibit stable convergence.

The total potential energy, total kinetic energy, and non-bonded energy all fluctuate within narrow ranges around their respective average values without unidirectional drift (Figure 1a), verifying energetic convergence of the system. The system temperature oscillates randomly around the target 298 K with a relative fluctuation of less than ±2.5% (Figure 1b), indicating well-controlled thermal conditions. The density undergoes rapid initial adjustment and then converges to a steady plateau (Figure 1c), confirming that the interchain packing structure has stabilized. For the single-chain conformation, the radius of gyration decreases sharply in the first few hundred picoseconds as the initial configuration relaxes, and subsequently levels off with only minor oscillations (Figure 1d), demonstrating complete conformational relaxation free of initial configuration bias. Corresponding equilibrium verification results for the other five systems are provided in Figures S1–S3 of the Supporting Information. All systems show convergence behaviors consistent with the A_20_ system, confirming that all constructed models have reached sufficient thermodynamic equilibrium and are reliable for subsequent mechanical and dynamic property simulations.

**Figure 2:**
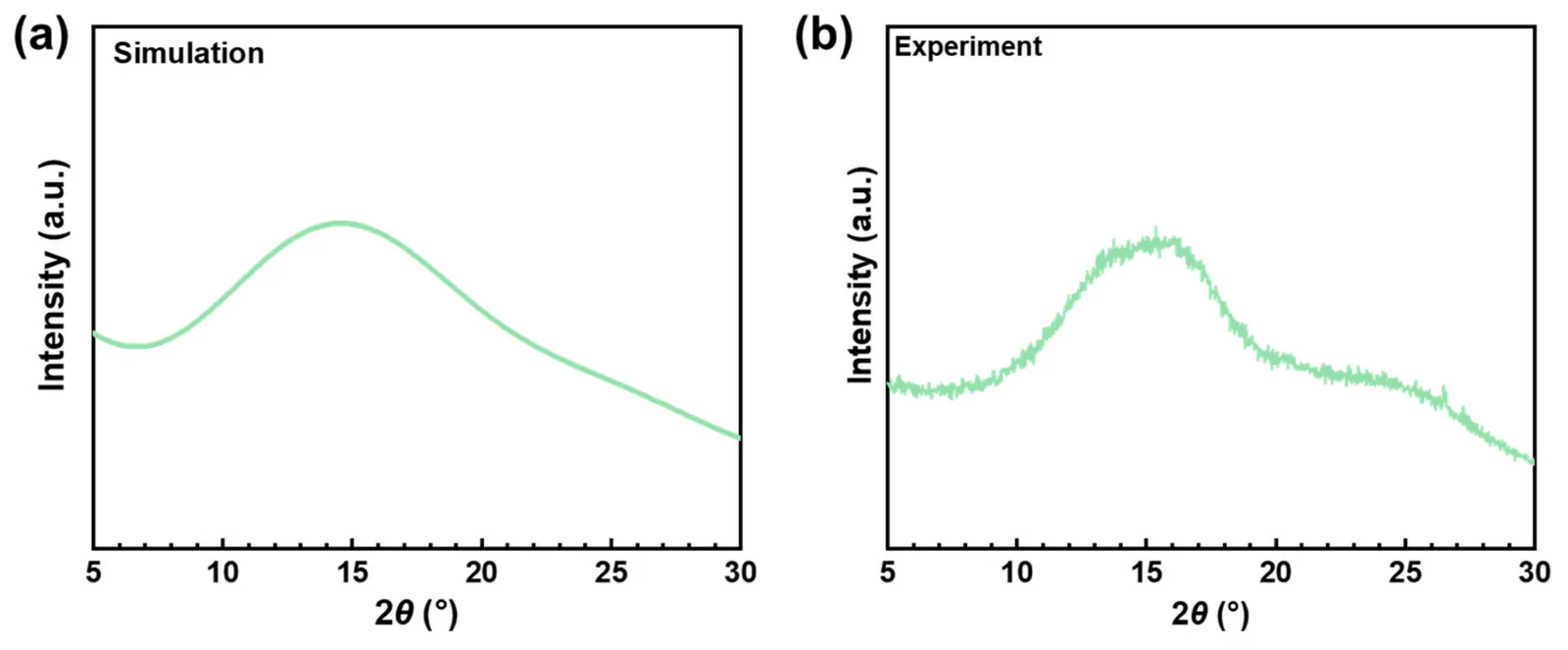
X-ray diffraction patterns of simulated A_20_ model (**a**), and experimental polyimide film (**b**) based on 6FDA/TFDB.

To further verify the physical representativeness of the equilibrated model, we compared the simulated X-ray diffraction pattern of the A_20_ model with the experimental result of the polyimide film based on 6FDA/TFDB, as shown in Figure 2. Both curves exhibit a single broad diffuse peak around 15° without sharp crystalline peaks, confirming that the simulated amorphous packing structure is consistent with the bulk experimental sample.

The quantitative comparison of key structural and mechanical parameters is summarized in Table 1. In terms of aggregation structure, the peak position deviation falls within the acceptable error range of all-atom molecular dynamics simulations. Additionally, the simulated density shows good consistency with the experimental value, and the overall agreement of structural parameters demonstrates that the packing state of the equilibrated model can reasonably reflect the aggregation structure of the real material.

In terms of mechanical properties, the simulated Young’s modulus and the experimental tensile modulus are in the same order of magnitude. It is commonly observed in all-atom molecular dynamics simulations of glassy polymers that the simulated modulus is moderately higher than the experimental value. This difference arises from the inherent distinctions between simulation and experiment: the simulated nanoscale model is an ideal defect-free amorphous system, free from the microscopic defects, voids, and structural heterogeneities that universally exist in macroscopic samples, and this idealized nature of the model leads to a moderately higher predicted modulus. This level of agreement between simulation and experiment confirms that the mechanical response of the model is physically reasonable.

The above experimental verifications, combined with the multi-parameter equilibration convergence analysis, jointly demonstrate that the established model can reliably reflect the intrinsic structural and mechanical properties of the material. Since the core of this work focuses on the relative effects of sequence structure on mechanical performance, this level of model accuracy provides a solid and valid basis for the subsequent comparative study across different copolymer systems.

### 2.2. Prediction of Microstructural Characteristics

#### 2.2.1. Effect of Sequence Structure on Molecular Chain Conformation

**Figure 3:**
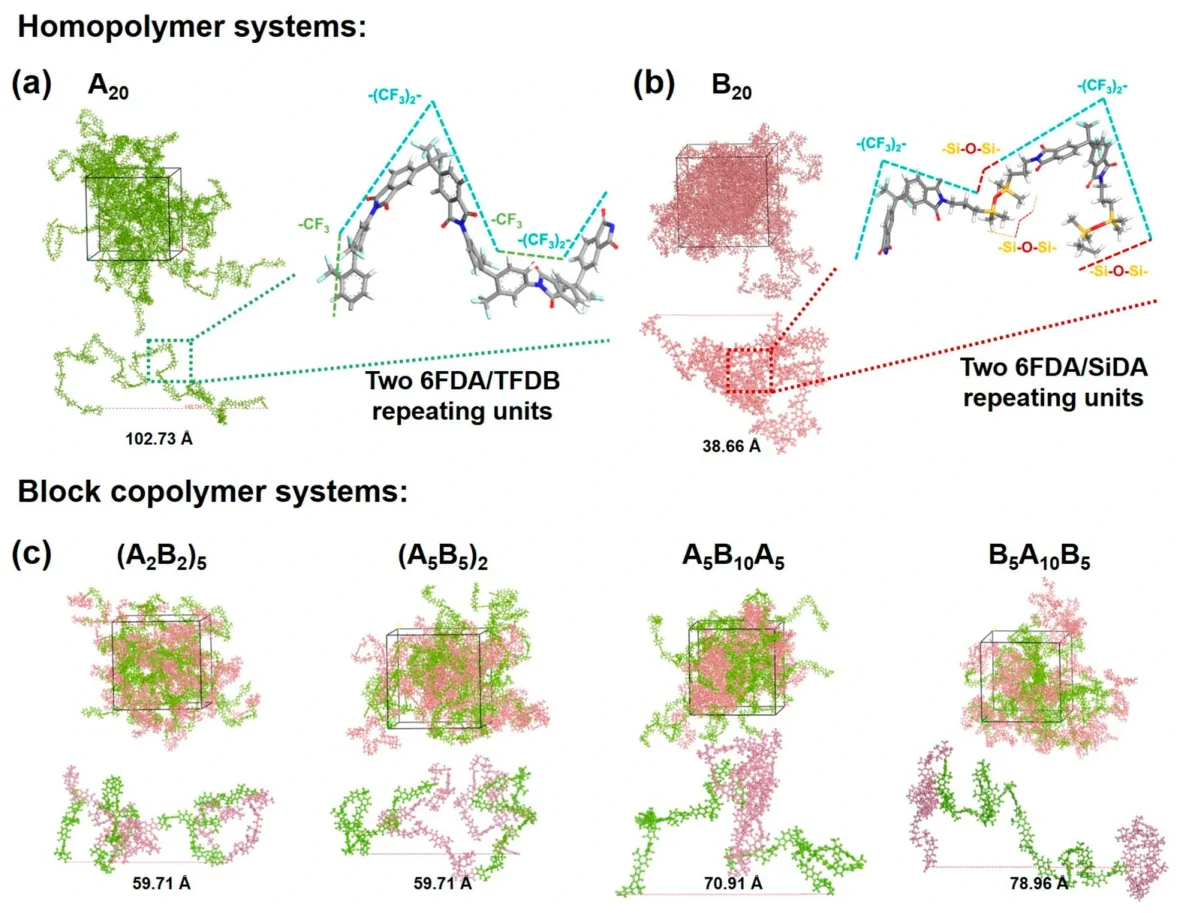
Equilibrated molecular models and representative single-chain conformations of the polyimides: A_20_ system (**a**), B_20_ system (**b**), and block copolymer systems (**c**). The insets show enlarged local conformations of the repeating units.

The equilibrated molecular models and representative single-chain conformations of the polyimides are shown in Figure 3. For each system, the displayed chain is the one whose mean-square end-to-end distance is nearest to the ensemble average. To the right of the molecular models in Figure 3a,b are the enlarged conformations of two repeating units extracted from the representative molecular chains, which are used to investigate the effects of main chain structure and side group types on molecular chain conformations. The conformational simulation parameters of the two homopolymer systems and the four block copolymer systems are listed in Table 2.

For the homopolymer systems, the single-chain conformation of the A_20_ system exhibits a Z-shaped zigzag folding conformation extending toward both ends. As revealed by the locally enlarged chain conformation, this mainly originates from the significant steric hindrance of the hexafluoroisopropyl group (blue dashed line) in dianhydride 6FDA. Such a Z-shaped extended conformation gives the A_20_ system the largest mean-square radius of gyration (⟨*s*^2^⟩), mean-square end-to-end distance (⟨*r*^2^⟩), and Kuhn segment length (*l*_b_), with values of 1515 Å^2^, 10,665 Å^2^, and 22.52 Å, respectively. In contrast, the B_20_ system exhibits a random coil conformation. This arises primarily from the continuous bending induced cooperatively by the hexafluoroisopropyl group in 6FDA and the Si-O-Si bond angle (red dashed line) in the SiDA diamine. As a result, the B_20_ system shows the smallest ⟨*s*^2^⟩, ⟨*r*^2^⟩, and *l*_b_ values of 357 Å^2^, 1589 Å^2^, and 3.00 Å, respectively.

**Figure 4:**
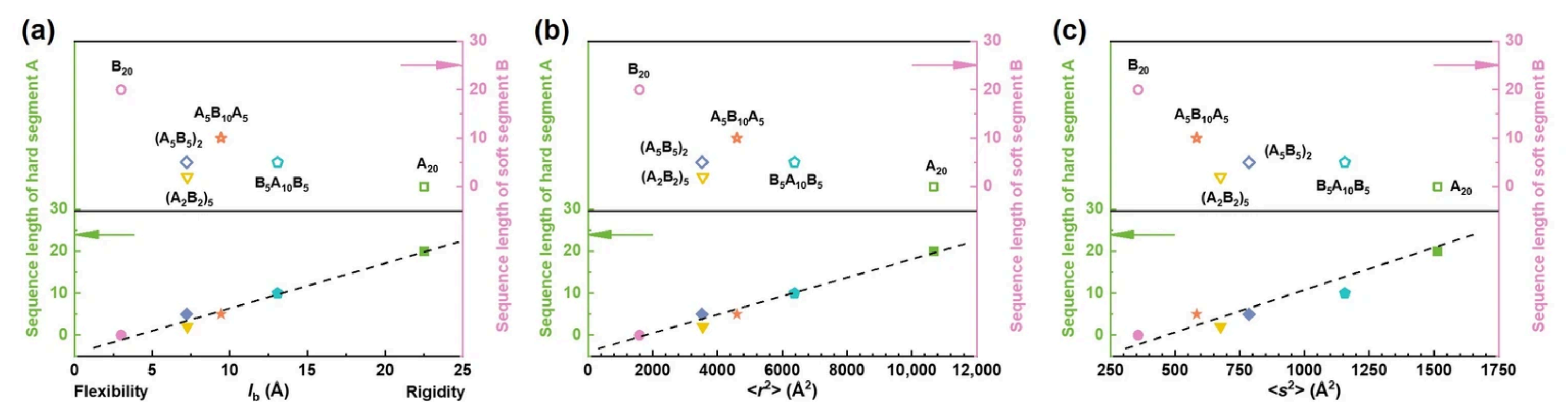
Relationships of *l*_b_ (**a**), ⟨*r*^2^⟩ (**b**), and ⟨*s*^2^⟩ (**c**) with the hard and soft segment sequence lengths.

For the four block copolymers, *l*_b_ and ⟨*r*^2^⟩ follow the same order: B_5_A_10_B_5_ > A_5_B_10_A_5_ > (A_2_B_2_)_5_ > (A_5_B_5_)_2_. This is because *l*_b_ is calculated from Equation (1), and the product *nl*_0_ is identical for all four copolymers; hence, *l*_b_ depends on ⟨*r*^2^⟩. A larger *l*_b_ value indicates higher chain rigidity. As shown in Figure 4a,b, the values of *l*_b_ and ⟨*r*^2^⟩ increase with the sequence length of hard segment A but show little correlation with the sequence length of soft segment B. This is because both ends of the soft segment are constrained by the rigid hard segment, which restricts the internal rotation of Si-O bonds and suppresses the flexibility of free siloxane chains. As a result, increasing the soft segment sequence length only slightly reduces overall chain rigidity, and the continuous hard segment A length remains the dominant factor controlling chain rigidity, with a far greater influence on *l*_b_ than soft segment B. Figure 4c shows that ⟨*s*^2^⟩ also increases with the sequence length of hard segment A. However, the A_5_B_10_A_5_ system exhibits the smallest ⟨*s*^2^⟩ in the block copolymer systems, despite having a relatively long hard segment sequence length. This is because ⟨*s*^2^⟩ is determined jointly by the overall chain extension and the spatial mass distribution. In the A_5_B_10_A_5_ system, the hard segments are located at both chain ends and adopt extended conformations, but each segment is of limited length. The long central soft segments adopt random coils, concentrating mass near the center of mass. It drastically reduces the average distance of atoms from the center of mass and ultimately yields the smallest ⟨*s*^2^⟩. In contrast, in the B_5_A_10_B_5_ system, the long hard segments are concentrated in the central region and form a rigid extended backbone. This gives the chain the highest degree of extension along the principal axis, while the terminal soft segments extend outward along the axis. Consequently, the average distance of atoms from the center of mass increases significantly, producing the largest ⟨*s*^2^⟩. It is suggested that the continuous hard segment length governs the chain extension capability and serves as the primary factor controlling molecular dimensions. The spatial distribution of hard segments further modulates the overall molecular size by altering the spatial accumulation of mass.

#### 2.2.2. Effect of Sequence Structure on Molecular Chain Mobility

**Figure 5:**
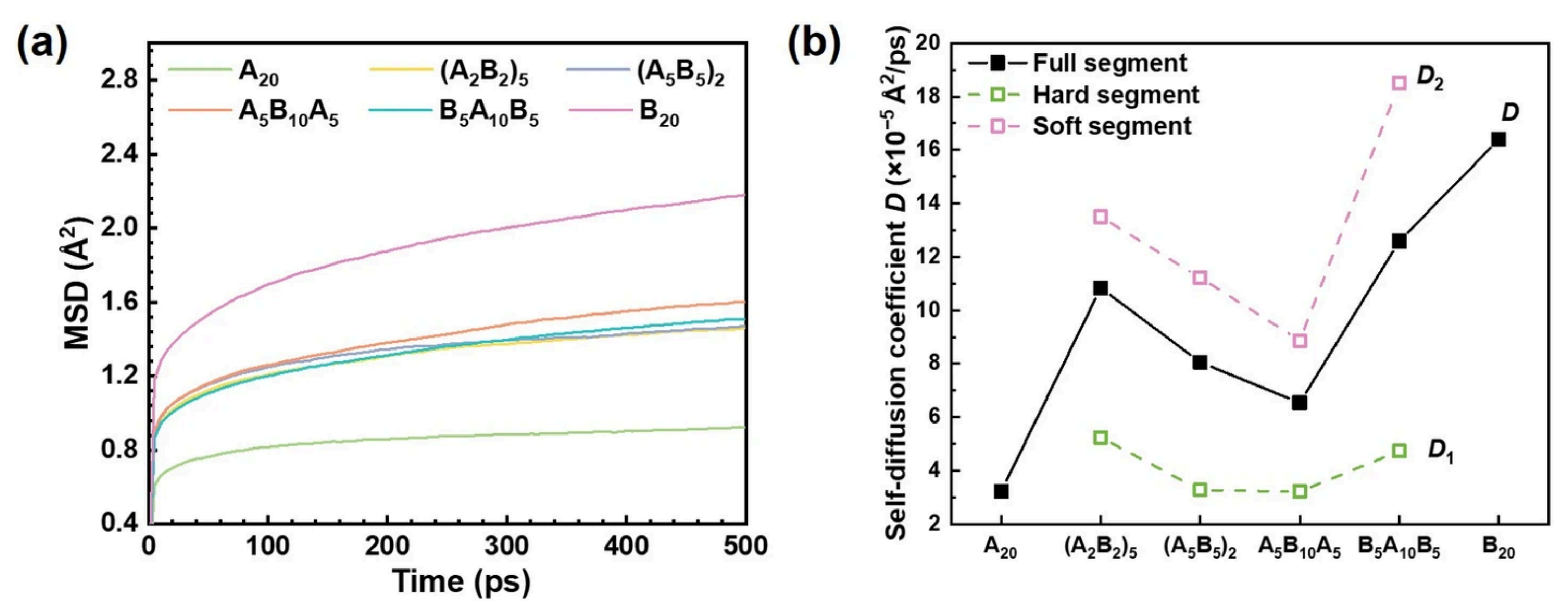
Simulated mean square displacement (MSD) curves (**a**) and self-diffusion coefficients *D* of the polyimide systems (**b**).

The effect of sequence structure on chain mobility was investigated by analyzing the mean square displacement (MSD) of the polyimide model using MD simulation. The simulated MSD curves and calculated self-diffusion coefficients (*D*) of these polyimide systems are presented in Figure 5a,b. For the homopolymer systems, the A_20_ system exhibits a *D* value of only 3.23 × 10^−5^ Å^2^/ps, indicating the lowest molecular chain mobility. In contrast, the B_20_ system shows a much higher *D* value of 16.40 × 10^−5^ Å^2^/ps, suggesting the soft segment imparts excellent mobility to the chains. For the block copolymers, the self-diffusion coefficient of the soft segments *D*_2_ follows the same trend as the total self-diffusion coefficient *D*, both in the order: B_5_A_10_B_5_ > (A_2_B_2_)_5_ > (A_5_B_5_)_2_ > A_5_B_10_A_5_. Moreover, *D*_2_ is much larger than that of the hard segments *D*_1_. This indicates that the soft segments contribute the major part of the chain mobility.

**Figure 6:**
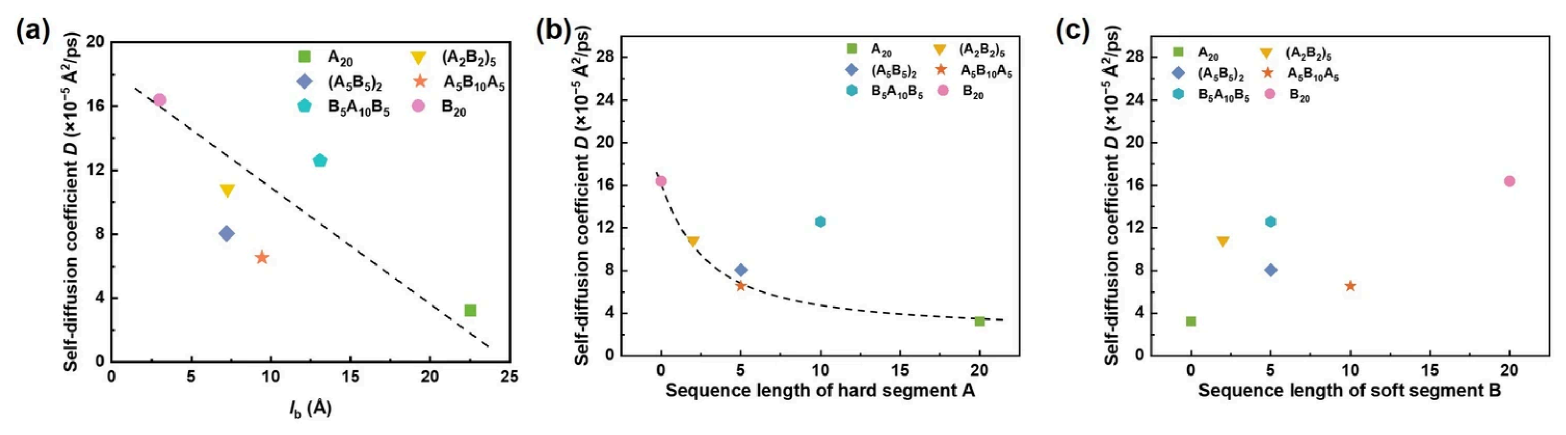
Relationship between *l*_b_ and self-diffusion coefficient *D* (**a**), relationships of *D* with sequence length of hard segment A (**b**), and sequence length of soft segment B (**c**).

Figure 6a compares the Kuhn segment length *l*_b_ with *D*. It is revealed that the *D* values gradually decrease as the *l*_b_ values increase. However, the B_5_A_10_B_5_ system, despite its high *l*_b_, exhibits a very high *D* value. It is supposed that molecular chain mobility is not solely determined by chain flexibility. Moreover, the *D* values generally decrease as the sequence length of the hard segment increases, except for the B_5_A_10_B_5_ system, whereas there is no obvious correlation between the *D* values and the sequence length of soft segments, as shown in Figure 6b,c. For the B_5_A_10_B_5_ system, the hard segments are concentrated in the central region of the chain and form only one aggregation center. This arrangement lacks constraints at both ends. Consequently, it exhibits the highest *D* value of 12.59 × 10^−5^ Å^2^/ps although it possesses the longest hard segment sequence. In contrast, the A_5_B_10_A_5_ system shows the opposite behavior. Its central soft segments are severely restricted by the aggregation-induced constraints from the hard segments at both ends, resulting in the lowest *D* value of 6.54 × 10^−5^ Å^2^/ps. When comparing the alternating systems of (A_2_B_2_)_5_ and (A_5_B_5_)_2_, it is found that the hard segments in the former are too short to form effective aggregation constraints, giving a relatively high *D* of 10.84 × 10^−5^ Å^2^/ps. The hard segments in the latter are long enough to establish effective constraints that restrict soft segment motion, yielding an intermediate *D* of 8.06 × 10^−5^ Å^2^/ps. These results suggest that molecular chain mobility is jointly governed by the formation of physical aggregates (determined by hard segment length) and their spatial distribution (determining constraint strength).

#### 2.2.3. Effect of Sequence Structure on Aggregation Structure

The chemical structure, molecular chain regularity, and flexibility of polymers all affect their aggregation structures, which in turn determine the macroscopic mechanical properties of materials. The effect of sequence structure on the aggregation state of polyimide molecular models was characterized by fractional free volume (FFV) and X-ray diffraction (XRD).

FFV is an important parameter for evaluating the space available for chain motion in polyimide models [36]. The calculated FFV values for each system are listed in Table 3. For the homopolymers, the A_20_ system shows the highest FFV of 13.50%. This arises from the bulky steric hindrance of trifluoromethyl groups and the bending effect of hexafluoroisopropyl groups, which together suppress close chain packing. The B_20_ system exhibits the lowest FFV of 6.93% because the flexible siloxane soft segments easily adopt random coil conformations, leading to denser chain packing. The FFV values of the four block copolymers range from 8.06% to 8.84%, falling between those of the two homopolymer systems.

**Figure 7:**
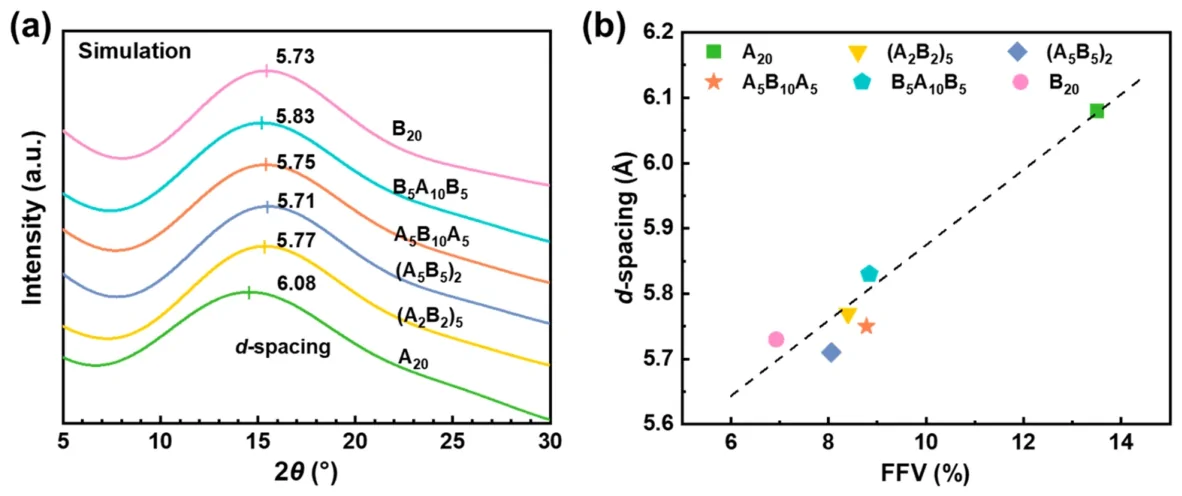
X-ray diffraction (XRD) patterns of the polyimide systems (**a**), and the relationship between *d*-spacing and FFV (**b**).

The molecular chain packing was further characterized by XRD, as shown in Figure 7a. All polyimide systems display broad diffraction peaks in the range of 14.55–15.50°, confirming their amorphous nature. As shown in Figure 7b, the interchain spacing (*d*-spacing) shows a positive correlation with FFV values. The trend observed for the homopolymer systems is fully consistent with the FFV results. The A_20_ system has a larger *d*-spacing, while B_20_ has a relatively smaller one. Among the block copolymers, the (A_5_B_5_)_2_ system has the smallest *d*-spacing. This is because the alternating arrangement of hard and soft segments forms a large helical conformation, which enhances interchain packing. The B_5_A_10_B_5_ system exhibits the highest *d*-spacing among the block copolymers. The mechanism is similar to that of the A_20_ system. The centrally aggregated long hard segments hinder close packing through the steric hindrance and bending effect of fluorine-containing groups.

**Figure 8:**
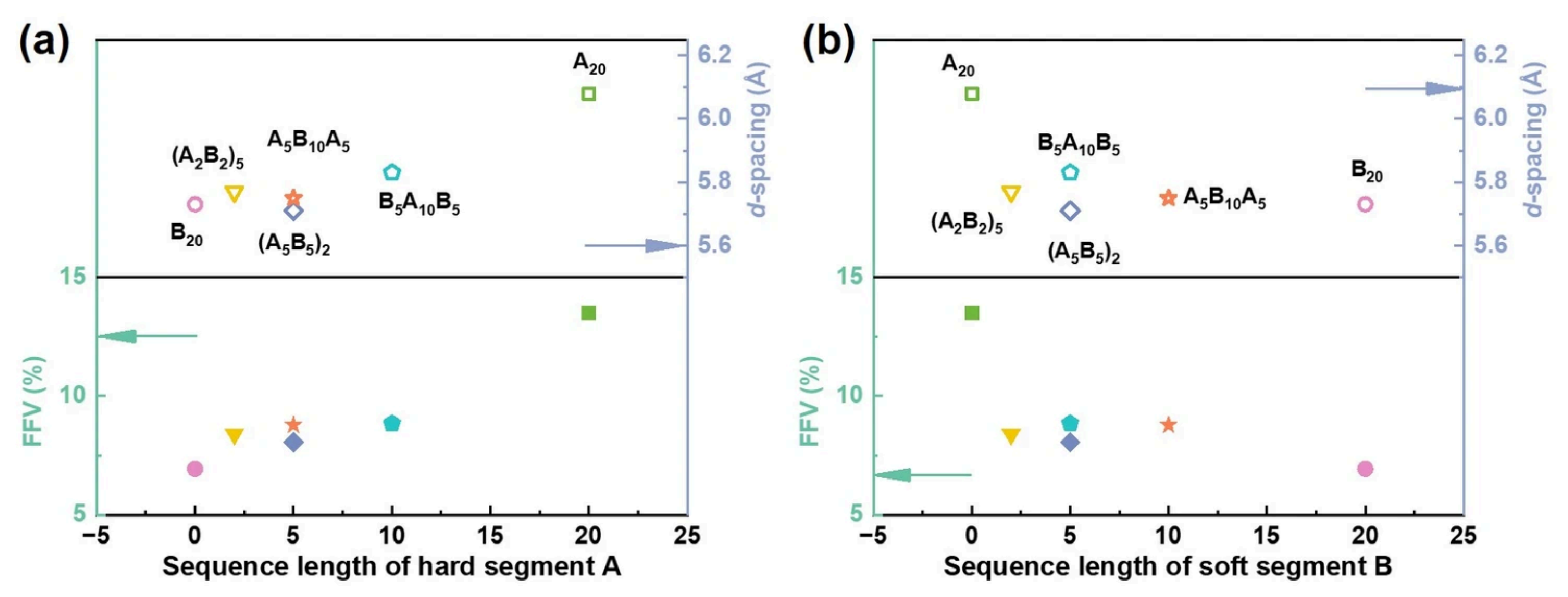
Relationships of FFV and *d*-spacing with hard segment sequence length (**a**) and soft segment sequence length (**b**).

Figure 8 further illustrates the overall influence of hard and soft segment sequence lengths on FFV and *d*-spacing. The results reveal that the two parameters differ only slightly among the four block copolymers. Moreover, both values are significantly lower than those of the A_20_ system. This indicates that the introduction of soft segments is the primary cause for the reduction in free volume and interchain spacing. In contrast, the sequence arrangement of soft segments has a relatively weak effect on the packing of the aggregation structures.

### 2.3. Prediction of Mechanical Properties

Mechanical properties serve as a core indicator for evaluating the applicability of materials. To investigate how sequence structure regulates the mechanical properties of polyimides, uniaxial tensile molecular dynamics simulations were performed on each molecular model along the X direction.

**Figure 9:**
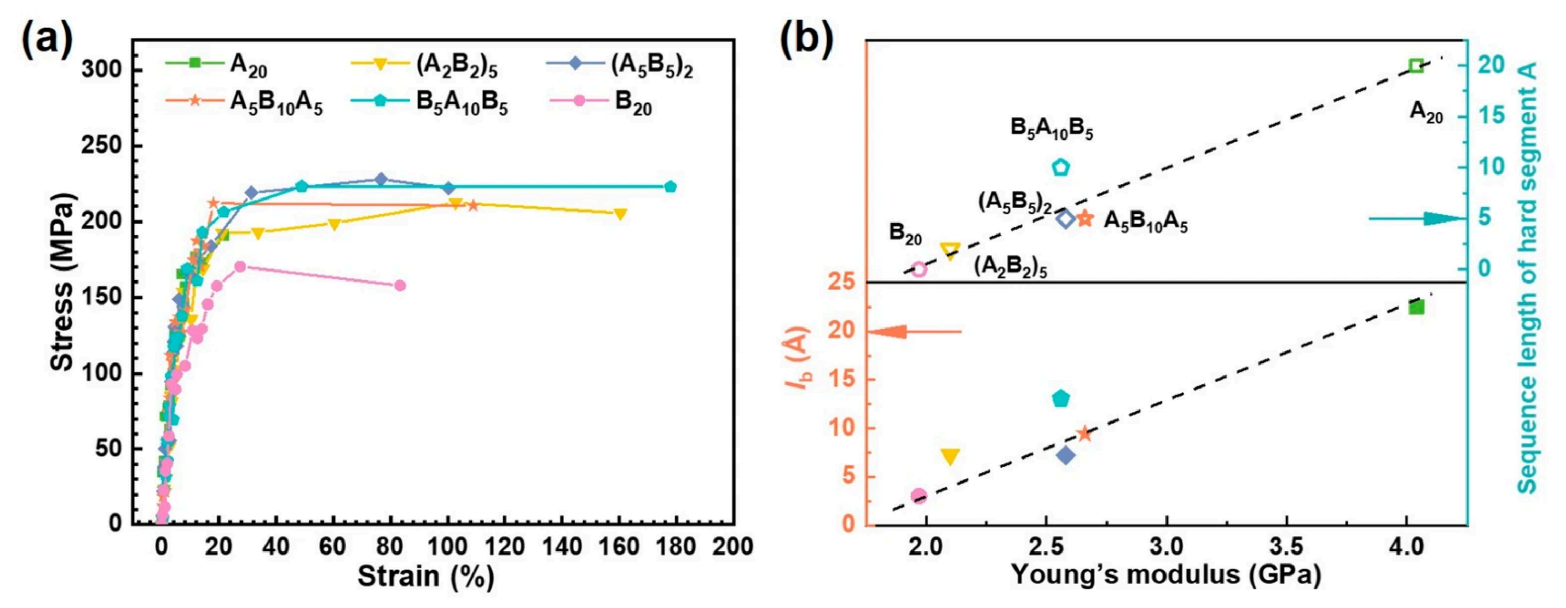
Stress–strain curves of the polyimide systems (**a**), and the relationships of Young’s modulus with *l*_b_ and hard segment sequence length (**b**).

The stress–strain curves of all systems are presented in Figure 9a. The Young’s modulus values calculated from these curves are shown in Figure 9b. The results indicate that the Young’s modulus of the six systems is positively correlated with the Kuhn segment length *l*_b_ and the hard segment sequence length. The A_20_ system exhibits the highest modulus of 4.04 GPa. This is attributed to its highest chain rigidity and longest hard segment sequence. The B_20_ system shows the lowest modulus of 1.97 GPa due to its lowest chain rigidity and the absence of continuous hard segments. The Young’s modulus of the four block copolymers falls between those of the two homopolymers.

**Figure 10:**
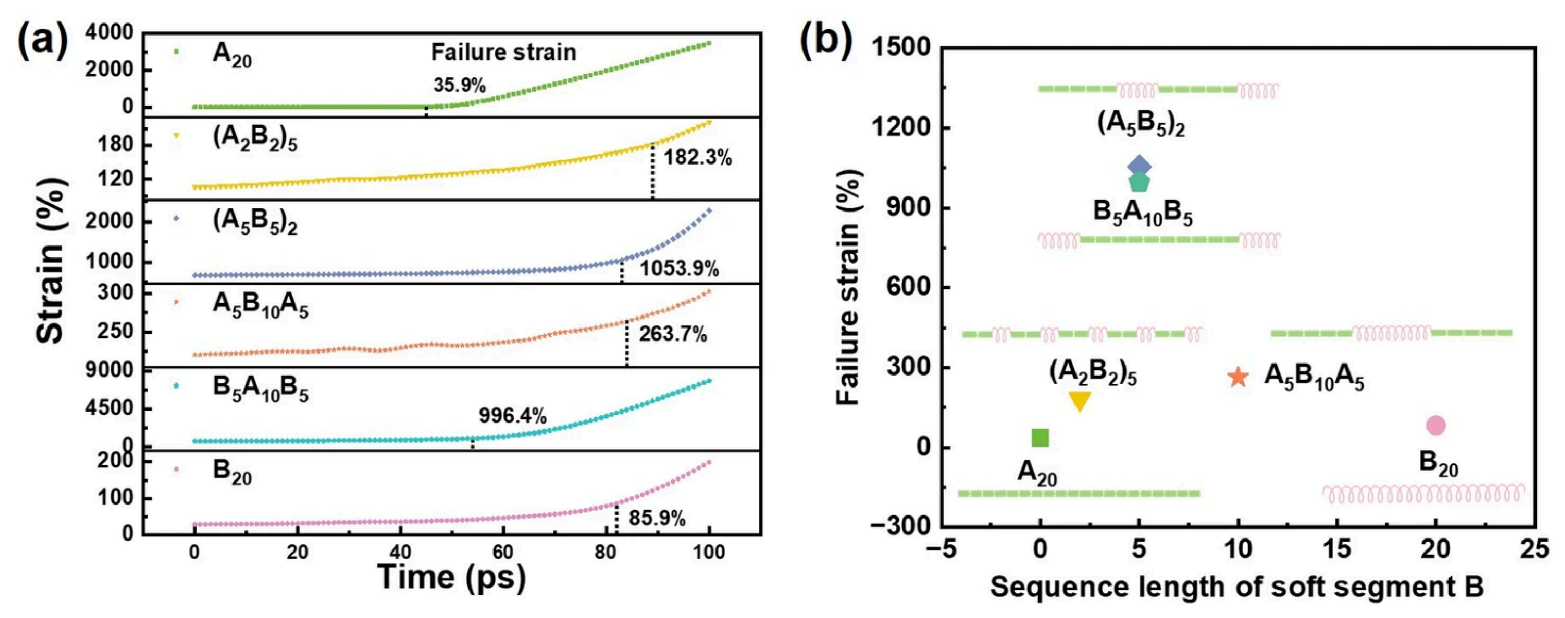
Comparison of failure strains of the polyimide systems (**a**), and the relationship between soft segment sequence length and failure strain (**b**).

The onset of the rapid strain increase after yielding was defined as the failure strain, which characterizes material toughness. The comparison results are shown in Figure 10a. The failure strains of the six systems range from 35.9% to 1053.9%. The overall order is (A_5_B_5_)_2_ > B_5_A_10_B_5_ > A_5_B_10_A_5_ > (A_2_B_2_)_5_ > B_20_ > A_20_. The relationship between soft segment sequence length and failure strain was further examined, as shown in Figure 10b. The failure strain first increases and then decreases as the soft segment length increases. The (A_5_B_5_)_2_ and B_5_A_10_B_5_ systems exhibit the relatively higher failure strains of 1053.9% and 996.4%, respectively. This is likely due to the moderate length of the soft segment, which enables effective stress dissipation through rapid conformational adjustment, significantly enhancing the failure strain. In contrast, the B_20_ system shows a much lower failure strain. This may be attributed to the excessively long soft segments, which cause chain entanglement and stress concentration and thus reduce the failure strain. Therefore, a moderate soft segment sequence length can improve chain flexibility while avoiding the negative effects induced by excessive entanglement.

### 2.4. Prediction of Tensile Failure Behavior

**Figure 11:**
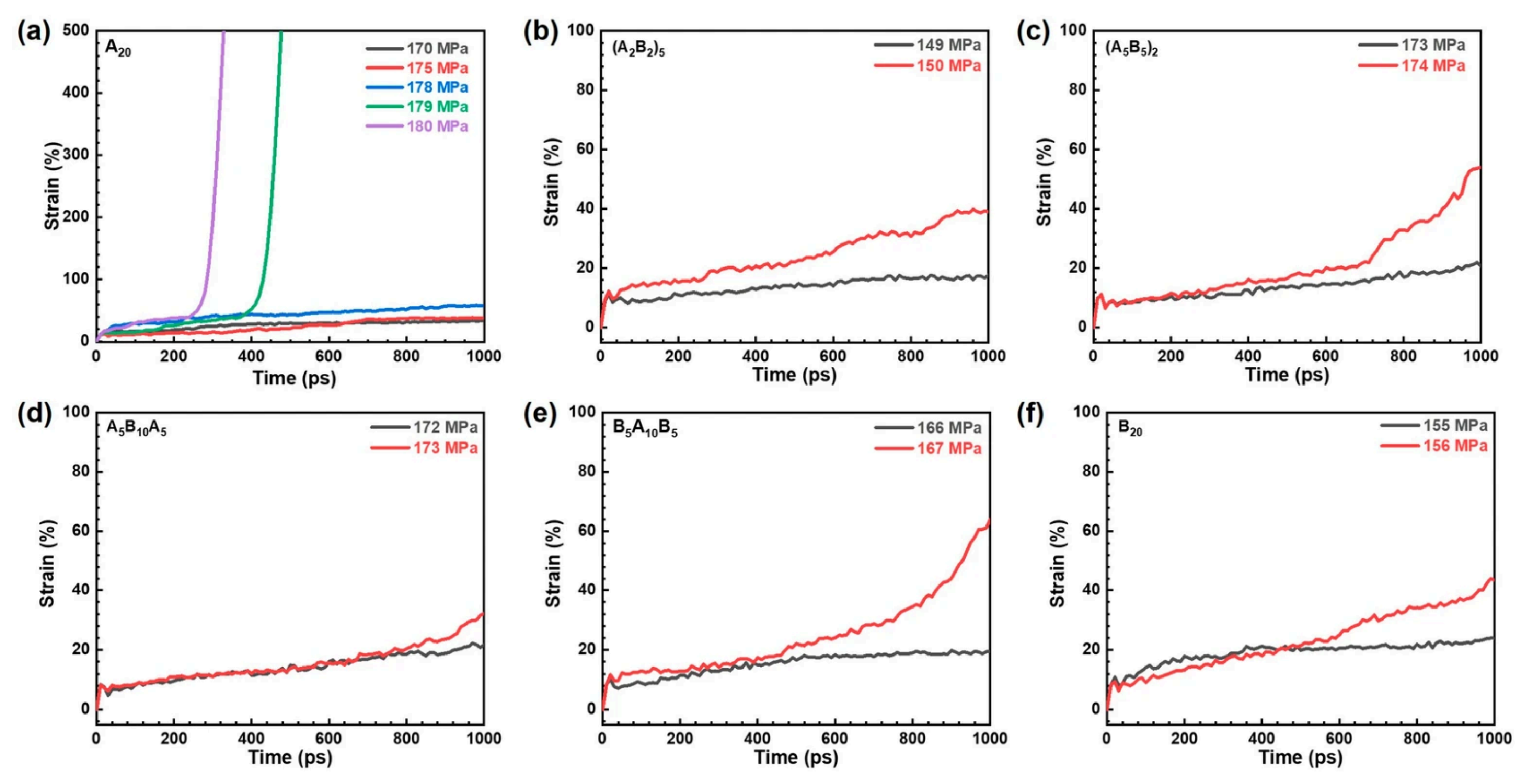
Creep curves of A_20_ system (**a**), (A_2_B_2_)_5_ system (**b**), (A_5_B_5_)_2_ system (**c**), A_5_B_10_A_5_ system (**d**), B_5_A_10_B_5_ system (**e**), and B_20_ system (**f**) at different stresses.

The stress threshold of a polymer is the critical stress that separates secondary creep from tertiary creep. Accurate determination of this value is essential for evaluating the stability and reliability of materials in practical applications [37,38]. In this study, the stress threshold was determined based on the stress near the yield point of the stress–strain curves. Creep simulations were performed to judge the occurrence of tertiary creep, and the bisection method was used to gradually narrow the failure stress range. The creep curves and stress threshold results for all systems are shown in Figure 11.

**Figure 12:**
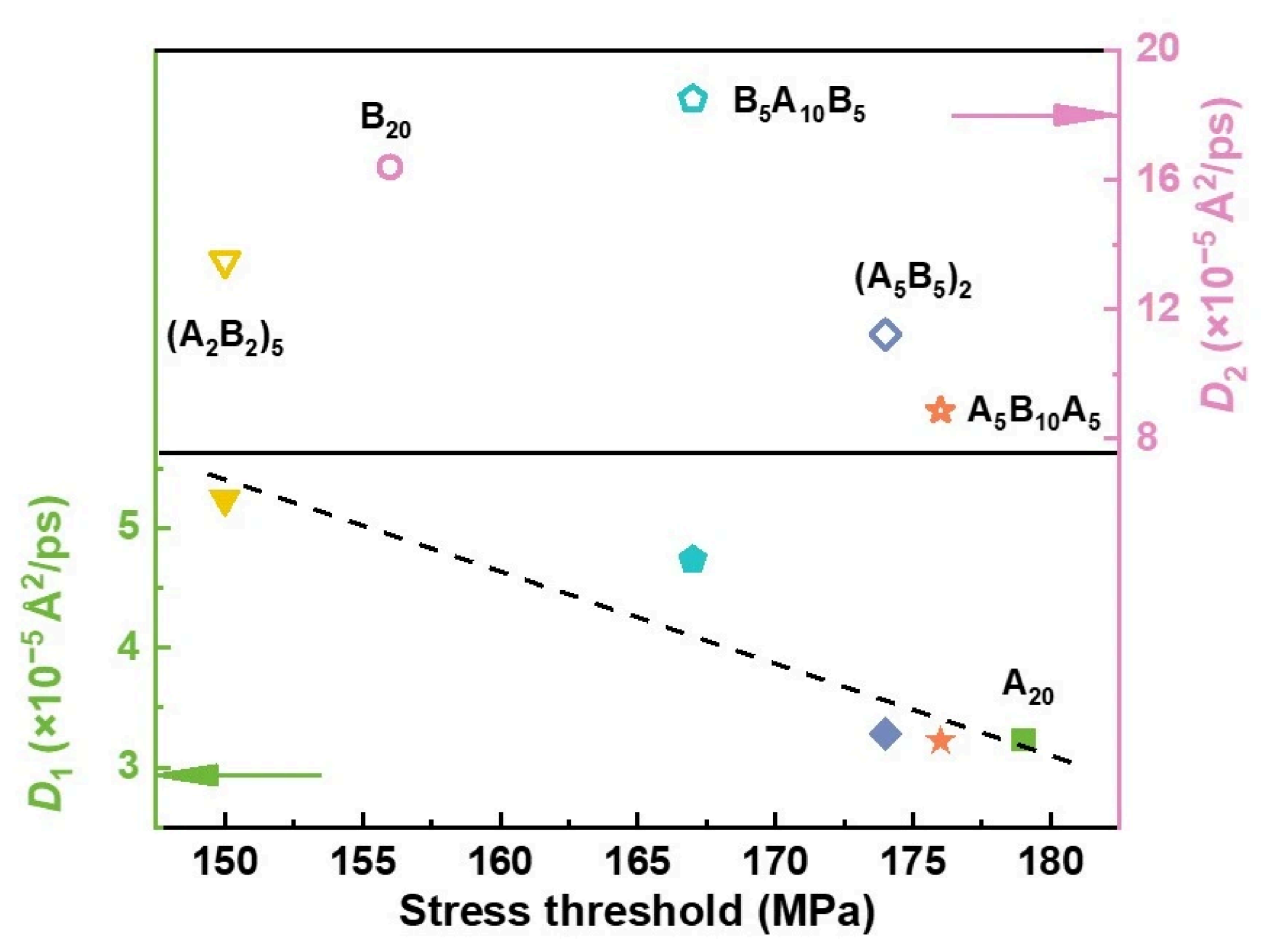
Relationships of stress threshold with the self-diffusion coefficients *D*_1_ (hard segments) and *D*_2_ (soft segments).

Taking the A_20_ system as an example, only secondary creep was observed at 170 MPa, whereas tertiary creep appeared at 180 MPa. The stress threshold was finally determined to be 179 MPa through iterative approximation. The stress thresholds for block copolymer systems are as follows: 150 MPa for (A_2_B_2_)_5_, 174 MPa for (A_5_B_5_)_2_, 173 MPa for A_5_B_10_A_5_, 167 MPa for B_5_A_10_B_5_, and 156 MPa for B_20_. The relationships between the self-diffusion coefficients of hard and soft segments and the stress threshold are shown in Figure 12. As the hard segment self-diffusion coefficient *D*_1_ increases, the stress threshold generally shows a decreasing trend. There is no consistent correlation between the soft segment self-diffusion coefficient *D*_2_ and the stress threshold. This indicates that hard segments play a dominant role in resisting plastic deformation.

**Figure 13:**
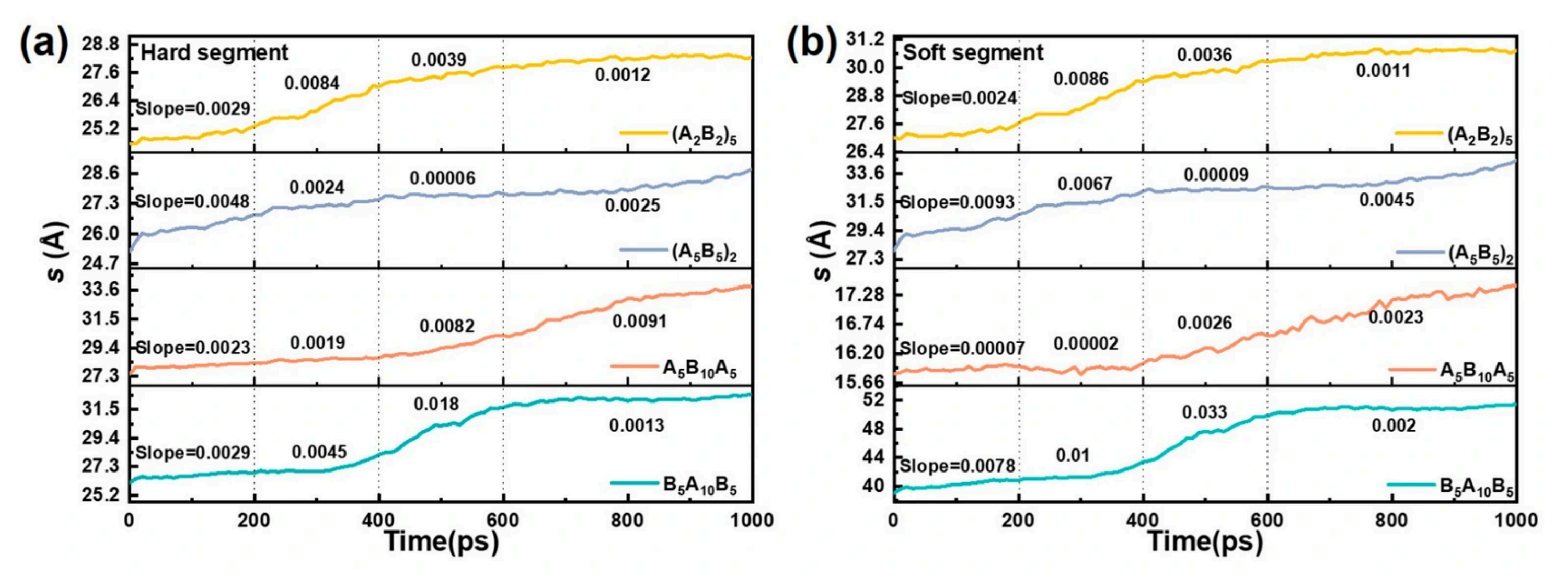
Evolution of the radius of gyration (*s*) for hard segments (**a**) and soft segments (**b**) under a constant stress of 180 MPa.

Since the stress thresholds of all systems range from 150 to 179 MPa, a constant stress of 180 MPa was uniformly selected to simulate the failure behavior. The evolution rates of the radius of gyration (*s*) for the hard and soft segments were analyzed to reveal the conformational changes during failure. The results are shown in Figure 13.

An increase in *s* corresponds to chain extension, while a decrease corresponds to chain coiling. The slope of the *s* curve reflects the rate of conformational change. A larger slope indicates a faster conformational transition, and a smaller slope indicates a slower change. For the four block copolymers, the differences in failure behavior can be explained by the variations in the *s* slopes of the hard and soft segments. For the (A_5_B_5_)_2_ and B_5_A_10_B_5_ systems, the *s* slope of the soft segments is approximately twice that of the hard segments during 0–200 ps, indicating that the soft segments undergo more rapid conformational changes. This can be interpreted as the soft segments effectively dissipate stress through rapid conformational adjustments, thereby avoiding local stress concentration. Both the (A_5_B_5_)_2_ and B_5_A_10_B_5_ systems exhibit relatively higher failure strains. In contrast, for the (A_2_B_2_)_5_ system, the *s* slopes of the hard and soft segments are highly synchronized. The rates of conformational change are the same, indicating that stress is uniformly distributed along the entire chain. However, the hard segment sequence length is too short to form effective physical aggregates for storing elastic energy. The chain cannot maintain its structural stability after deformation, resulting in the lowest failure strain.

### 2.5. Prediction of Creep Performance

**Figure 14:**
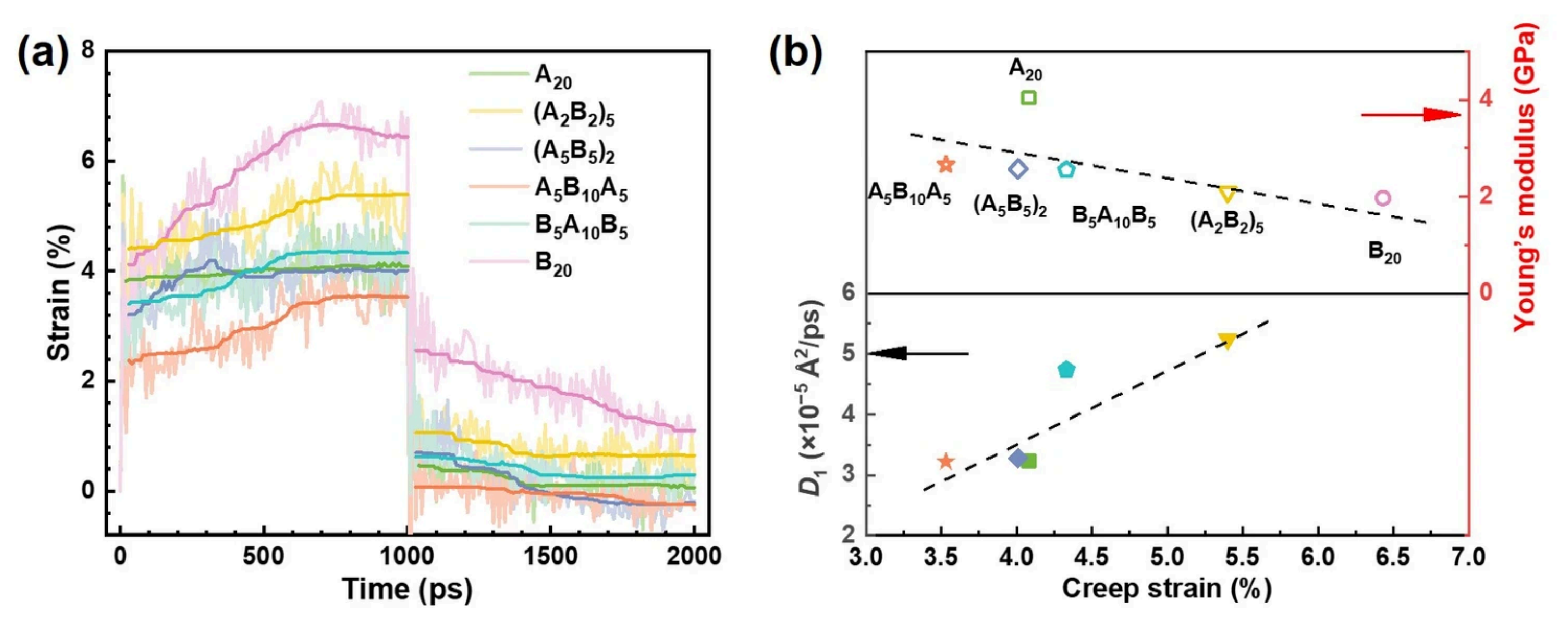
Creep curves of the polyimide systems (**a**), and the relationship of creep strain with *D*_1_ and Young’s modulus (**b**).

The polymeric materials applied as stretchable substrates for flexible wearable electronics must maintain their shape and mechanical properties over long periods to prevent damage to devices [39]. To evaluate the long-term reliability of the siloxane-containing polyimides, a constant stress of 100 MPa was selected for creep simulations. This stress corresponds to the minimum yield stress within 5% strain for all systems. The results are shown in Figure 14a. All four block copolymer systems exhibit typical three-stage creep behavior under the applied stress. The primary creep stage shows rapid initial deformation. The steady-state creep stage is characterized by a nearly constant deformation rate. After unloading, the creep recovery process consists of rapid elastic recovery followed by slow viscous recovery. The creep performance differs significantly among the block copolymer systems with different sequence structures. The creep strain ranges from 3.53% to 5.40% and increases in the order of A_5_B_10_A_5_ < (A_5_B_5_)_2_ < B_5_A_10_B_5_ < (A_2_B_2_)_5_. The (A_5_B_5_)_2_ and A_5_B_10_A_5_ systems exhibit creep recovery rates as high as 100%. The (A_2_B_2_)_5_ and B_5_A_10_B_5_ systems give the relatively lower creep recovery rates of 87.96% and 93.07%, respectively.

The relationship between creep strain, Young’s modulus, and the hard segment self-diffusion coefficient *D*_1_ is further illustrated in Figure 14b. The creep strain decreases as the Young’s modulus increases. In contrast, the creep strain increases as *D*_1_ increases. This indicates that the hard segments play a dominant role in resisting creep deformation.

**Figure 15:**
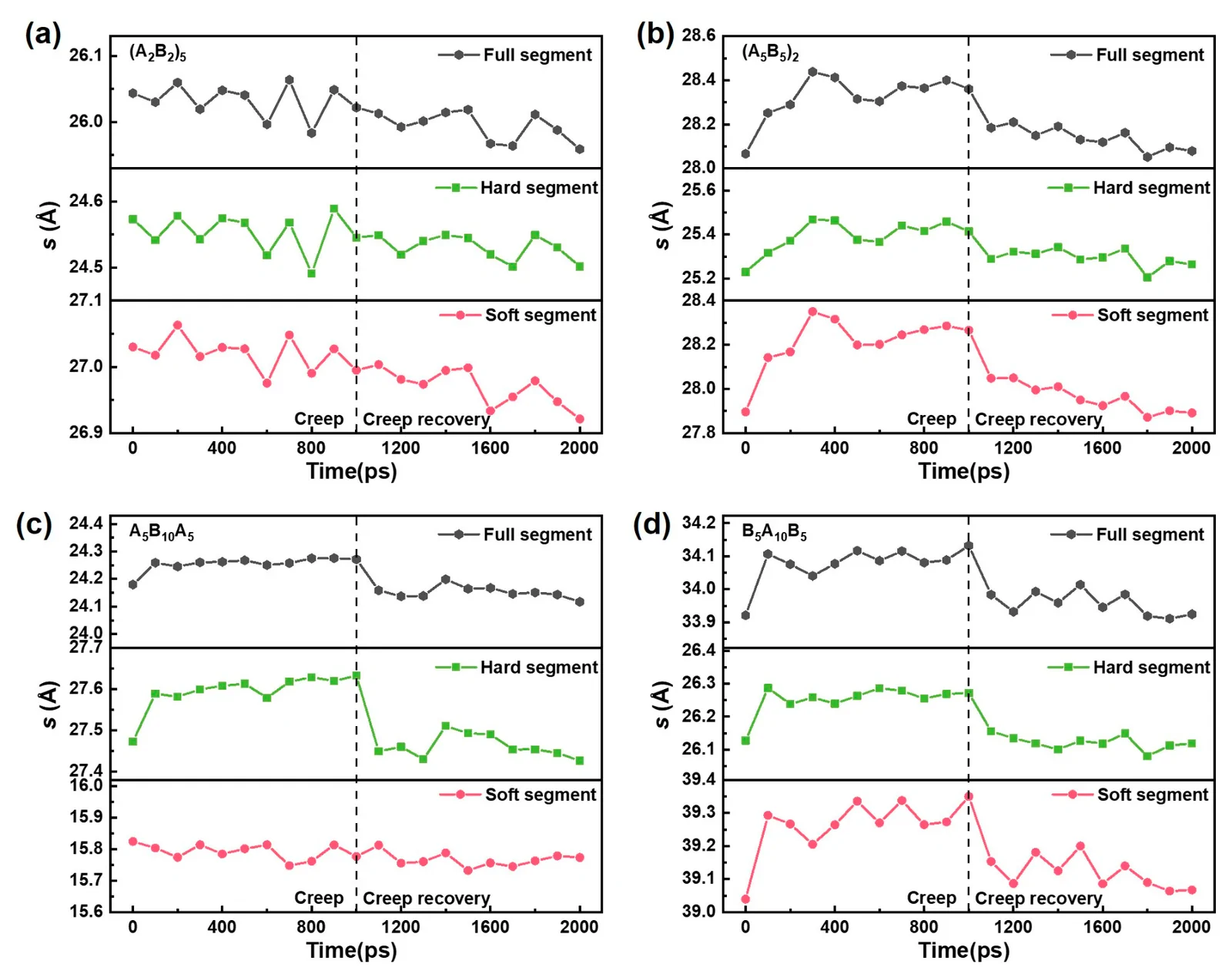
Evolution of the radius of gyration (*s*) of hard segments, soft segments, and the total chains during creep and recovery for the (A_2_B_2_)_5_ (**a**), (A_5_B_5_)_2_ (**b**), A_5_B_10_A_5_ (**c**), B_5_A_10_B_5_ (**d**) polyimide systems.

**Figure 16:**
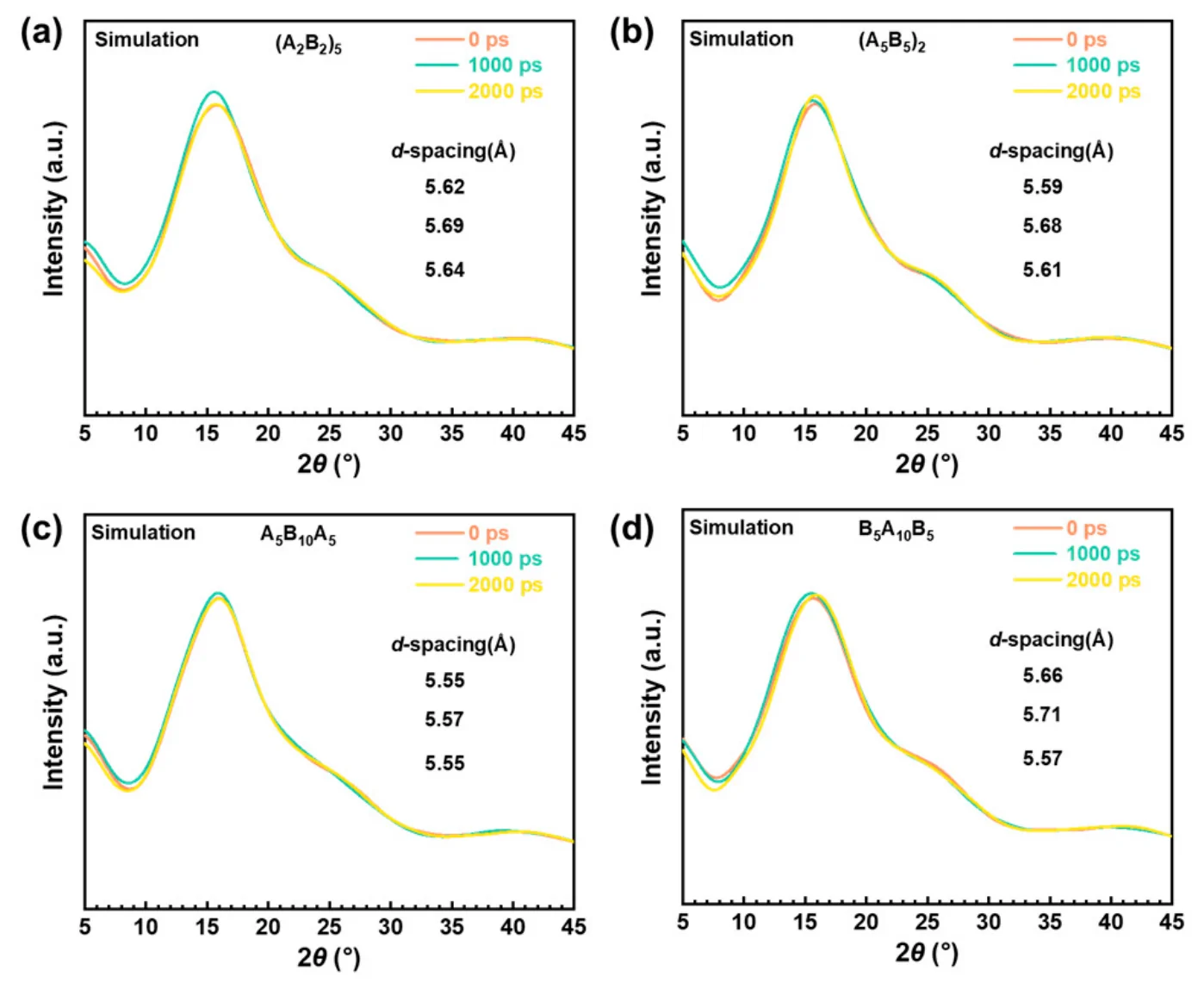
XRD patterns of the (A_2_B_2_)_5_ (**a**), (A_5_B_5_)_2_ (**b**), A_5_B_10_A_5_ (**c**), B_5_A_10_B_5_ (**d**) systems at the initial (0 ps), after creep (1000 ps), and after recovery (2000 ps) stages.

**Figure 17:**
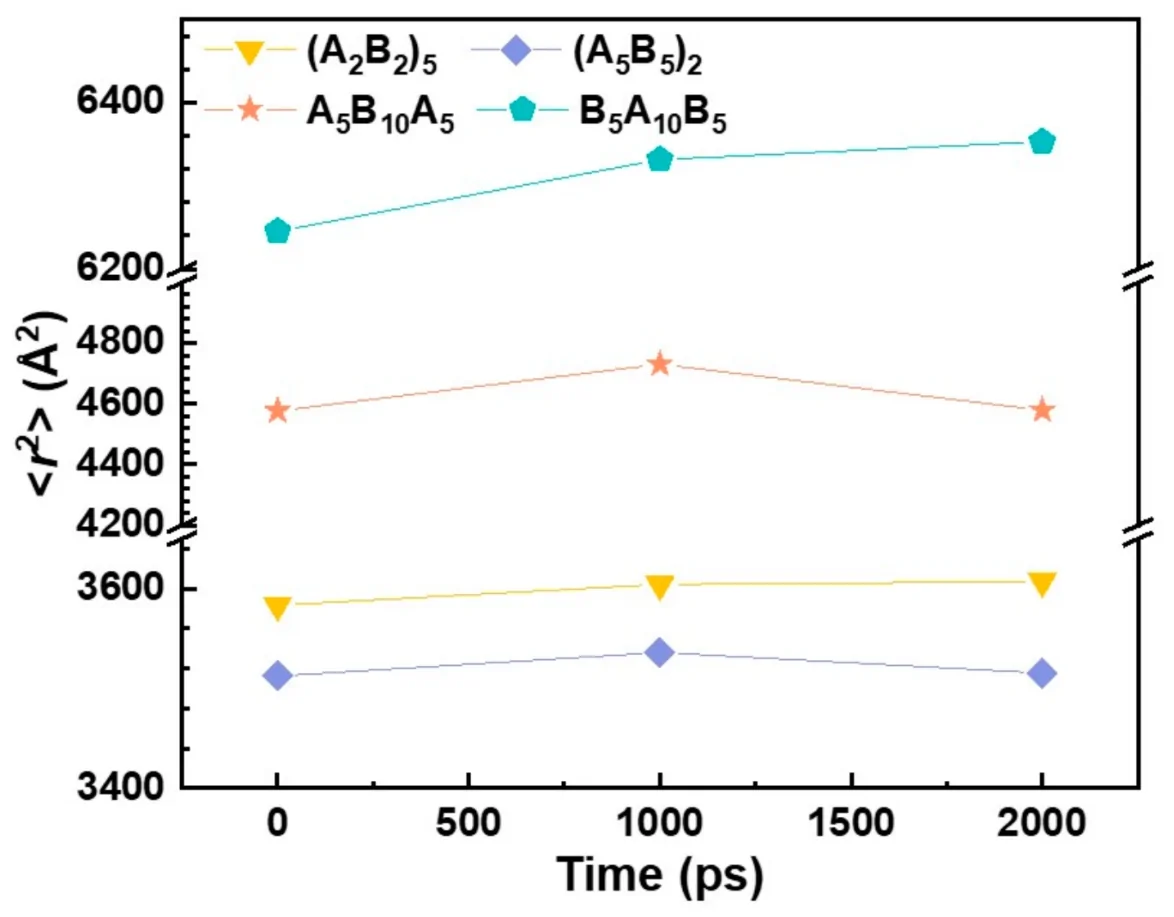
Mean-square end-to-end distance ⟨*r*^2^⟩ of the block copolymer systems at the initial, after creep, and after recovery stages.

To reveal the microscopic evolution process during creep and recovery, the changes in chain conformation and aggregation structure were analyzed at three key stages. These stages are the initial state at 0 ps, the end of creep at 1000 ps, and the state after full recovery at 2000 ps. The analyzed parameters include the radius of gyration (*s*), aggregation structures, and mean-square end-to-end distance (⟨*r*^2^⟩) illustrated in Figure 15, Figure 16 and Figure 17. The corresponding results of the main XRD peak position (2*θ*) and intensity, and the standard deviation of the center-of-mass distribution (*σ*_COM_) are listed in Table 4.

**Table 4:** Main XRD peak position (2*θ*) and intensity, and standard deviation of the center-of-mass distribution (*σ*_COM_) of the block copolymer systems at 0, 1000, and 2000 ps.

PI	0 ps			1000 ps			2000 ps		
	2*θ* (°)	Intensity (a.u.)	*σ*_COM_	2*θ* (°)	Intensity (a.u.)	*σ*_COM_	2*θ* (°)	Intensity (a.u.)	*σ*_COM_
(A_2_B_2_)_5_	15.75	94.46	15.61	15.55	97.49	15.65	15.70	94.71	15.59
(A_5_B_5_)_2_	15.85	94.80	14.41	15.60	95.82	14.41	15.80	97.22	14.37
A_5_B_10_A_5_	15.95	94.65	14.41	15.90	96.22	14.41	15.95	94.46	14.37
B_5_A_10_B_5_	15.65	93.08	14.70	15.50	94.59	14.74	15.90	94.07	14.71

During the creep stage, all systems show a shift in the main XRD peak to lower angles and an increase in peak intensity, indicating an increase in interchain spacing. The changes in *s*, ⟨*r*^2^⟩, and *σ*_COM_ differ significantly among the systems. For the (A_2_B_2_)_5_ system, *s* and ⟨*r*^2^⟩ remain almost unchanged during creep, but *σ*_COM_ along the tensile direction increases. This indicates that creep deformation originates from relative interchain slippage. In the recovery stage, the interchain spacing, main peak intensity, and *σ*_COM_ largely return to their initial values while *s* decreases as inner chain segments retract elastically toward the chain center of mass and ⟨*r*^2^⟩ shows a slight increase from residual slippage at chain ends instead of full recovery. This is because the hard segment sequence is too short to form effective physical aggregates to anchor the chain segments. Therefore, the driving force for recovery is insufficient after unloading, leading to the highest creep strain and the lowest creep recovery rate. For the (A_5_B_5_)_2_ system, *s* of both hard and soft segments, as well as ⟨*r*^2^⟩, increase during creep, while *σ*_COM_ remains unchanged. This indicates that the deformation is the conformational stretching of the chains themselves. After unloading, these parameters recover almost completely. However, the intensity of the XRD peak shows a further increase, and *σ*_COM_ becomes smaller than the initial value. This suggests that the hard segment aggregates reorganize into a more ordered and denser state after unloading, resulting in a creep recovery rate of 100%. For the A_5_B_10_A_5_ system, the soft segment *s* remains nearly unchanged during creep, whereas the hard segment *s* and ⟨*r*^2^⟩ increase. The XRD peak position and intensity change only slightly, and *σ*_COM_ remains constant. This demonstrates that the deformation is mainly contributed by the conformational stretching of the hard segments at both ends. The central soft segments are strongly constrained and do not participate in the deformation. After unloading, the hard segment conformations return completely, and *σ*_COM_ becomes smaller than the initial value. Therefore, a creep recovery rate of 100% is also achieved. For the B_5_A_10_B_5_ system, *s*, ⟨*r*^2^⟩, and *σ*_COM_ increase during creep. This reflects the asymmetric conformational extension of the central hard segments and the terminal soft segments. In the recovery stage, the hard segment *s* recovers almost completely, but the soft segment *s* does not fully retract. The ⟨*r*^2^⟩ remains higher than the initial values, and the XRD peak shifts to higher angles, exceeding the initial position. This indicates that after recovery, the chains form a more ordered parallel alignment along the tensile direction. Although the recovery is not complete, a relatively high creep recovery rate is achieved.

**Figure 18:**
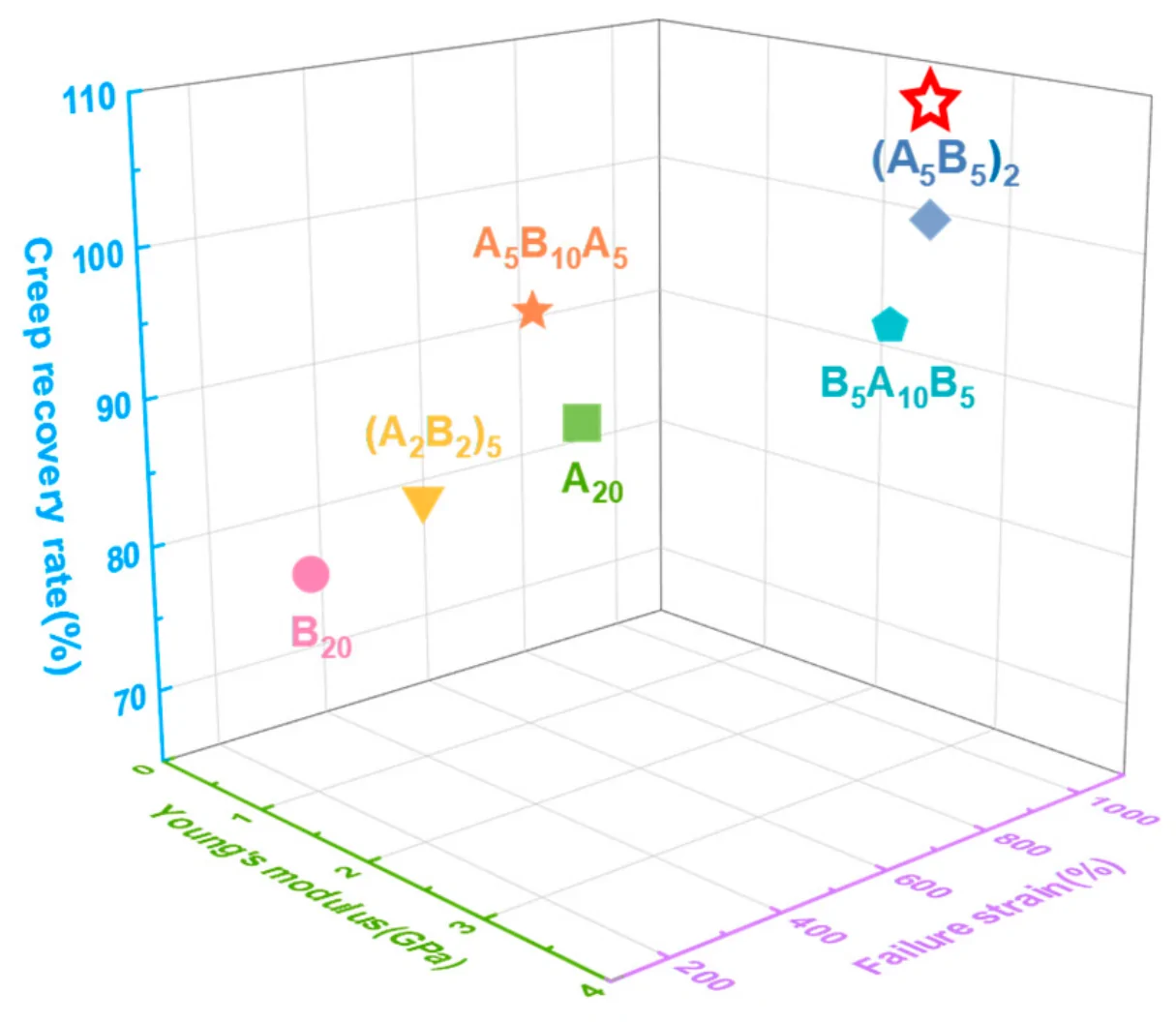
Comprehensive comparison of Young’s modulus, failure strain, and creep recovery rate for the polyimide systems.

Figure 18 compares six systems across three core indicators: Young’s modulus, failure strain, and creep recovery rate. The A_20_ system possesses the highest modulus but the lowest failure strain and only moderate creep recovery, while the B_20_ system shows poor modulus and recovery performance despite high chain flexibility. Among the four block copolymers, the (A_5_B_5_)_2_ system achieves the highest failure strain and a 100% creep recovery rate, while maintaining a relatively high modulus. This well-balanced combination of stiffness, large deformation capacity, and long-term dimensional stability makes (A_5_B_5_)_2_ the optimal comprehensive performer among all systems.

## 3. Materials and Methods

### 3.1. Polymer Structure Design

**Scheme 1:**
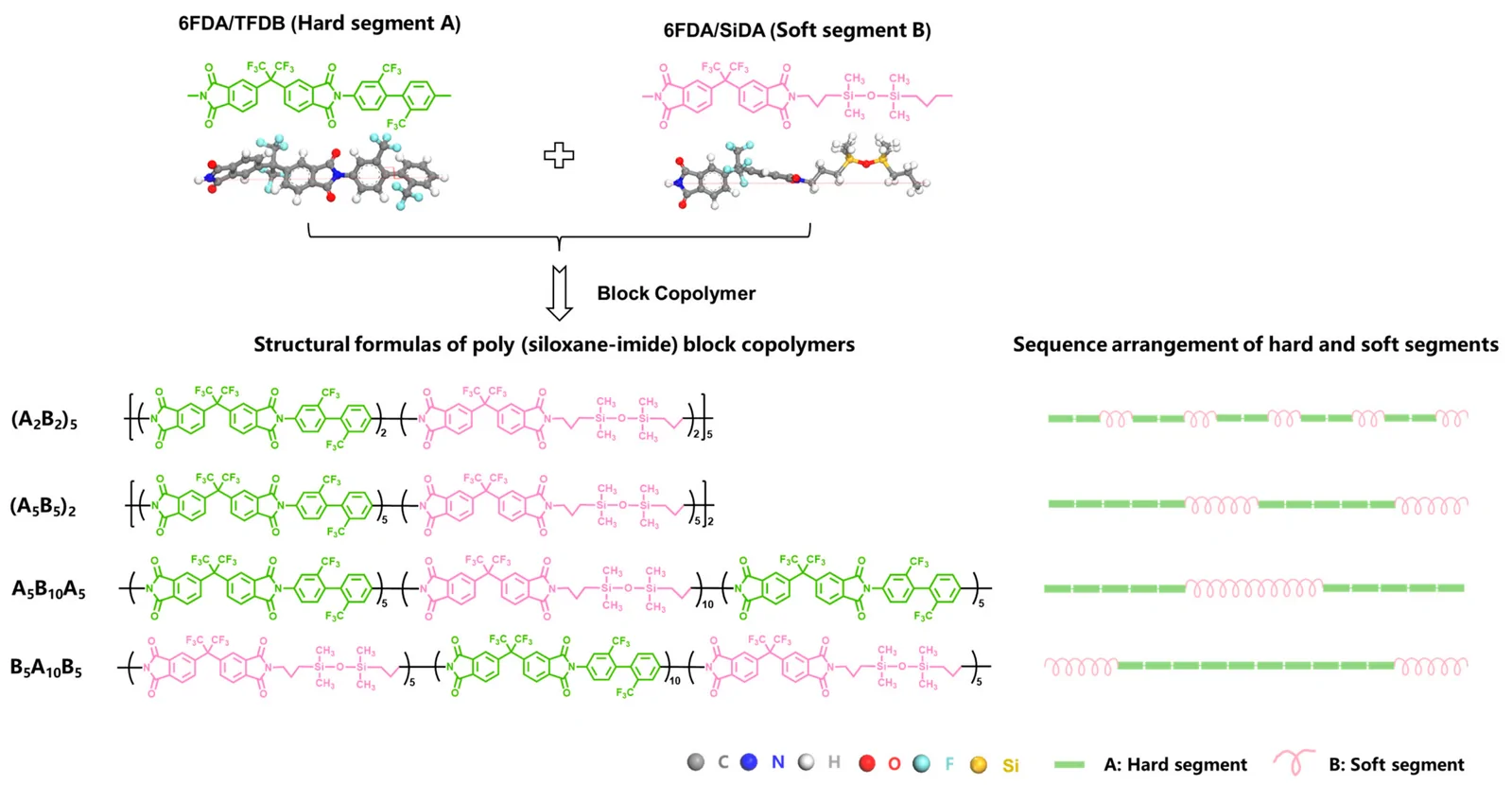
Structural formulas and sequence arrangement of hard and soft segments of poly(siloxane-imide) block copolymers.

A series of poly(siloxane-imide)s was designed based on fluorinated aromatic dianhydride 4,4′-(hexafluoroisopropylidene)diphthalic anhydride (6FDA), fluorinated aromatic diamine 2,2′-bis(trifluoromethyl)-4,4′-diaminobiphenyl (TFDB), and siloxane-containing diamine bis(4-aminophenoxy)dimethylsilane (SiDA). The sequential block polyimide copolymers consisting of the hard segment of 6FDA/TFDB and the soft segment of 6FDA/SiDA were designed with the structure as shown in Scheme 1. The 6FDA/TFDB and 6FDA/SiDA repeating units are defined as hard segment A and soft segment B, respectively. The ratio of hard segments A to soft segments B was designed to be 1:1, meaning that a single polyimide molecular chain comprises 10 hard segment As and 10 soft segment Bs. Based on the differences in the sequence lengths of the hard and soft segments, the designed sequence structures of the block copolymers are (A_2_B_2_)_5_, (A_5_B_5_)_2_, A_5_B_10_A_5_, and B_5_A_10_B_5_. The sequence lengths of the hard and soft segments in polyimide copolymer systems with different sequence structures are listed in Table 5.

### 3.2. Construction of Molecular Models

The structural models of polyimide molecular chains were constructed using BIOVIA Materials Studio 2019. Each cubic simulation cell contained 10 molecular chains, with each chain consisting of 20 repeating units. The COMPASS II force field was adopted for the simulations [40,41,42]. The quality was set to “Ultra-Fine”, the pressure to 0.0001 GPa, and the time step to 1.0 fs. Additionally, the van der Waals (vdW) interactions were calculated using the atom-based method, and the electrostatic interactions were calculated using the group-based method. The cubic simulation box of polyimide was constructed using the Amorphous Cell module. Setting the initial density of the simulation box to 0.1 g/cm^3^ can effectively avoid high-energy unstable conformations caused by atomic overlap and ensure reasonable initial packing of aromatic functional groups. The spatial geometry of the constructed cell model was optimized to find the lowest potential energy point. The Smart method with an energy convergence criterion of 2 × 10^−5^ kcal/mol and a maximum number of iterations of 10^5^ was used for geometric optimization to obtain the global minimum energy configuration. If the convergence condition was not met, the geometric optimization was repeated until the convergence condition was satisfied. To achieve a fully relaxed state of the box, annealing procedures were performed using the Anneal tool in the Forcite module: the system was subjected to a 20-cycle annealing process, heated from 300 K to 800 K at a rate of 5 K/ps, and then cooled to 300 K at the same rate. The full annealing was conducted sequentially under the NPT and NVT ensembles for 2000 ps each to locate the lowest-energy conformation. The high-temperature annealing step at 800 K, which is far above the glass transition temperature of the polyimides, enables full dihedral rotation and extensive chain rearrangement, thereby releasing the structural bias introduced by initial random packing. Subsequently, equilibrium molecular dynamics simulations were performed using the Dynamics tool in the Forcite module: First, a 2000 ps NPT ensemble simulation was performed, followed by a 2000 ps NVT ensemble simulation. The last 500 ps of trajectory data from each ensemble were used for subsequent analysis.

### 3.3. Characterization of Simulated Structures

The mean-square end-to-end distance ⟨*r*^2^⟩, mean-square radius of gyration ⟨*s*^2^⟩, and Kuhn segment length *l*_b_ are commonly used to characterize the conformational state of polymer molecular chains [43]. When the polymer chain is stretched or adopts a more extended conformation, its radius of gyration increases; conversely, when it is compressed or adopts a more coiled conformation, its radius of gyration decreases. A larger *l*_b_ value indicates higher molecular chain rigidity, while a smaller *l*_b_ value implies greater molecular chain flexibility. The formula for calculating *l*_b_ is as follows [44]:

(1) $$l_{b}=\frac{\left\langle r^{2}\right\rangle }{{nl}_{0}}$$

where ⟨*r*^2^⟩ denotes the mean square end-to-end distance of the molecular chain, *n* represents the number of repeating units in a single molecular chain, and *l*_0_ is the length of a single repeating unit in its fully extended state.

The mean square displacement (MSD) is defined as the ensemble average of the squared displacement of particles (or chain segments) relative to their initial positions over the observation time *t*. The formula for calculating MSD is as follows [26,45]:

(2) $$\mathrm{MSD}=\frac{1}{N}\sum_{i=1}^{N}\left\langle {\left|r_{i}\left(t\right)-r_{i}\left(0\right)\right|}^{2}\right\rangle$$

where *r_i_*(0) denotes the position vector of particle *i* at time *t* = 0, *r_i_*(*t*) denotes the position vector of particle *i* at time *t*, and *N* represents the total number of atoms in the system.

The self-diffusion coefficient *D* is a scalar quantity describing the rate of random diffusion of particles or chain segments in a medium. It is defined as the slope of the MSD curve in the long-time limit, which represents the molecular chain mobility, and is calculated according to Formula (3) [46]:

(3) $$D=\frac{1}{6}\underset{t\to \infty }{\lim}\frac{\mathrm{dMSD}}{d\Delta t}$$

The fractional free volume (FFV) was calculated using the Connolly surface method. FFV is defined as the ratio of the free volume to the total volume of the simulation cell, and its formula is as follows [47]:

(4) $$\mathrm{FFV}=\frac{V-V_{0}}{V}=\frac{V-1.3V_{w}}{V}$$

where *V* is the total volume of the equilibrated system, *V*_0_ is the occupied atomic volume, and *V*_W_ is the van der Waals volume.

X-ray diffraction (XRD) patterns of all PI systems were analyzed using the Analysis function in the Forcite module [48]. The radiation type was set to X-ray, the scattering cutoff distance was set to 12 Å, and the X-ray wavelength was 1.54178 Å (consistent with experimental values). The 2*θ* scanning range was 5–45°. All XRD patterns are simulated data from equilibrated all-atom models. For these fully amorphous copolymers, the *d*-spacing calculated by the Bragg equation refers to the statistically averaged interchain distance. The Bragg equation is expressed as follows:

(5) 2dsinθ=nλ

where *d* is the interplanar spacing, *θ* is the diffraction angle, *λ* is the X-ray wavelength, and *n* is the diffraction order.

Stress–strain curves were obtained by performing uniaxial tensile simulations along the X axis on each equilibrated model using the built-in stress–strain script in BIOVIA Materials Studio 2019 software [49,50]. First, each model was relaxed along the X axis for 500 ps, and 5 relaxation cycles were performed to eliminate residual stress in the X direction. Subsequently, tensile stress was applied along the X axis at increments of 10 MPa, with the stress in the other two directions maintained at 0 MPa. For each stress level, the model was first equilibrated for 50 ps, followed by a constant-stress tensile simulation for 50 ps. The actual stress and strain values recorded by the system were used to plot the stress–strain curves.

The yield point was defined as the point on the stress–strain curve where a stress plateau first appears, or the maximum stress was reached, and the corresponding stress value was designated as the yield strength. The Young’s modulus was calculated as the slope of the stress–strain curve in the 0–2% strain range. In the uniaxial tensile simulations, if the strain of a polyimide system exhibited a rapid increase with time under a certain stress after the yield point, the onset of this rapid increase was defined as the failure strain. This parameter is only used for comparative analysis between different simulation systems and cannot be equated to the experimental elongation at break.

Creep curves (time–strain curves) were obtained by stretching each model along the X axis under a constant stress, with the stress in the other two directions maintained at 0 MPa. Each simulation was run for 1000 ps in the NPT ensemble, and the creep curves were obtained by analyzing the change in the model length along the X axis as a function of time using the Analysis module [51]. The creep strain (*ε*_creep_) is defined as the total strain after 1000 ps under constant stress. The residual strain (*ε*_residual_) is defined as the strain remaining after releasing the constant stress applied in the X-direction to 0 and equilibrating for 1000 ps. The creep recovery rate is calculated as follows:

(6) $$\mathrm{Creep}\;\mathrm{recovery}\;\mathrm{rate}=\frac{\varepsilon_{\mathrm{creep}}-\varepsilon_{\mathrm{residual}}}{\varepsilon_{\mathrm{creep}}}\times 100\%$$

To quantitatively characterize the spatial dispersion of molecular chain centers of mass, we define the standard deviation of the center-of-mass distribution (*σ*_COM_). This parameter reflects the uniformity of chain distribution in space: a larger *σ*_COM_ indicates a more dispersed and random distribution of chain centers of mass, whereas a smaller *σ*_COM_ indicates a more concentrated and ordered arrangement. The *σ*_COM_ is calculated using the following formula:

(7) $$\sigma_{\mathrm{COM}}=\sqrt{\frac{1}{N}\sum_{i=1}^{N}{\left|r_{i}-\left\langle r\right\rangle \right|}^{2}}$$

where *N* is the total number of molecular chains in the simulation cell, *r_i_* is the position vector of the center of mass of the *i*-th chain, and ⟨*r*⟩ is the ensemble-average position vector of all chain centers of mass in the system.

### 3.4. Experimental and Characterization Methods

To validate the physical reliability and representativeness of the established all-atom simulation model, experimental characterization was performed on a 6FDA/TFDB homopolyimide sample having the same chemical structure as the A_20_ simulation model.

#### 3.4.1. Materials

6FDA was obtained from Sanming Hexafluo Chemicals Co., Ltd. (Fujian, China) and dried under vacuum at 120 °C for 12 h before use. TFDB was obtained from Changzhou Sunshine Pharmaceutical Co., Ltd. (Changzhou, China) and used as received. Anhydrous ethanol, N,N-dimethylacetamide (DMAc), acetic anhydride, and pyridine were purchased from Beijing Chemical Works (Beijing, China) and used without further purification.

#### 3.4.2. Synthesis of 6FDA/TFDB Polyimide Resin

The diamine TFDB (0.1 mol) and the solvent DMAc (244 mL) were added to a 1 L three-necked flask and stirred at room temperature for 0.5 h. After cooling to 0 °C in an ice-water bath, dianhydride 6FDA (0.1 mol) was added and fully dissolved under stirring. The ice bath was then removed, and the mixture was stirred at room temperature for 24 h to yield a highly viscous polyamic acid (PAA) solution with 25 wt% of solid content. Acetic anhydride (0.5 mol) and pyridine (0.25 mol) were then added and reacted for another 6 h to complete imidization. The resulting solution was poured into an ethanol/water mixture to give a white, fiber-like precipitate. The polyimide resin was obtained after being collected by filtration and dried in vacuum at 120 °C for 8 h.

#### 3.4.3. Preparation of 6FDA/TFDB Polyimide Film

The polyimide resin was dissolved in DMAc with stirring to form a homogeneous solution with a solid content of 15 wt%. The solution was filtered and degassed, and then cast onto a glass substrate using an automatic film applicator. The wet film was thermally baked at 80 °C for 2 h, followed by 250 °C for 1 h. After cooling to room temperature, the polyimide film was obtained by immersing the glass substrate in deionized water, followed by drying.

#### 3.4.4. Characterization of 6FDA/TFDB Polyimide Film

X-ray diffraction (XRD) measurement was performed on a PANalytical Empyrean-P2 diffractometer (Malvern Panalytical, Almelo, Netherlands) with Cu Kα radiation (*λ* = 0.154 nm). The test was operated at a tube voltage of 40 kV and a tube current of 200 mA, with a 2*θ* scanning range of 5° to 40°. The interchain d-spacing of the amorphous polyimide film was calculated according to Bragg’s law Equation (5).

The Young’s modulus was tested according to GB/T 1040.3-2006 [52] using an LS5 material testing machine (Lloyd Instruments, Bognor Regis, UK) at a tensile rate of 10 mm/min. Five groups of valid data were averaged to obtain the final result.

The film density was determined by the immersion method in accordance with GB/T 1033.1-2008 [53]. Tests were conducted at 23 °C using deionized water as the immersion liquid. Each specimen was weighed in air and in the liquid in triplicate, and the density was calculated and averaged to three decimal places in g/cm^3^.

## 4. Conclusions

A series of poly(siloxane-imide) block copolymer models based on 6FDA/TFDB as hard segment A and 6FDA/SiDA as soft segment B with distinct sequence structures were investigated by molecular dynamics simulations. The effects of sequence structure, including block length and block arrangement pattern, on the microscopic chain conformation, molecular chain mobility, aggregation structure, as well as macroscopic tensile properties and creep behaviors of the copolymer systems, were clarified. The results suggested that the hard segment length and distribution jointly dictate chain dimensions and rigidity, and further regulate the aggregation state of the systems. Moreover, the chain mobility is not only determined by chain flexibility, but also strongly affected by the aggregation state of the hard segments. For macroscopic properties, failure strain is jointly regulated by the lengths of the hard and soft segments, where the hard segment length controls the formation of physical aggregates and the stress transfer pathway. The creep recovery behavior follows the same regulatory logic: stable physical aggregates inhibit irreversible interchain slippage and enable reversible conformational extension. Among all systems, the (A_5_B_5_)_2_ structure exhibits the best comprehensive performance. This is because its sufficiently long hard segments form effective physical aggregates to resist irreversible deformation, while the moderate soft segment length provides adequate stress dissipation without excessive entanglement. It is suggested that the alternating sequence ensures a uniform stress distribution and symmetric deformation. It should be noted that only one independent amorphous cell was simulated for each polymer composition in this study. While the large system size, multi-frame trajectory sampling, and rigorous multi-step annealing equilibration protocol have been employed to minimize statistical uncertainty, we acknowledge that replica-to-replica variations have not been explicitly quantified. Future work with multiple independent initial configurations will be carried out to further validate the quantitative precision of the results.

## Figures and Tables

**Table 1:** Structural and mechanical parameters of simulated A_20_ model and experimental polyimide film based on 6FDA/TFDB.

Parameter	Simulation	Experiment
Density (g/cm^3^)	1.465	1.461
XRD peak (2*θ*, °)	14.55	15.36
*d*-spacing (Å)	6.08	5.76
Young’s modulus (GPa)	4.04	3.66

**Table 2:** Conformational parameters of the polyimide systems ^a^.

PI	Density (g/cm^3^)	*nl*_0_ (Å)	⟨*s*^2^⟩ (Å^2^)	⟨*r*^2^⟩ (Å^2^)	*l*_b_ (Å)
A_20_	1.465	473	1515	10,665	22.52
(A_2_B_2_)_5_	1.400	488	676	3545	7.27
(A_5_B_5_)_2_	1.406	488	786	3521	7.22
A_5_B_10_A_5_	1.397	488	584	4600	9.44
B_5_A_10_B_5_	1.396	488	1156	6375	13.08
B_20_	1.294	530	357	1589	3.00

^a^ *nl*_0_: the length of 20 repeating units; ⟨*s*^2^⟩: mean-square radius of gyration; ⟨*r*^2^⟩: mean-square end-to-end distance; *l*_b_: Kuhn segment length.

**Table 3:** Total volume (*V*), occupied volume (*V*_0_), free volume (*V*_1_), and fractional free volume (FFV) of the polyimide systems.

PI	*V* (Å^3^)	*V*_0_ (Å^3^)	*V*_1_ (Å^3^)	FFV (%)
A_20_	165,117	142,825	22,292	13.50
(A_2_B_2_)_5_	164,251	150,452	13,799	8.40
(A_5_B_5_)_2_	163,656	150,460	13,196	8.06
A_5_B_10_A_5_	164,723	150,268	14,455	8.78
B_5_A_10_B_5_	164,782	150,222	14,560	8.84
B_20_	168,636	156,948	11,690	6.93

**Table 5:** Hard and soft segment sequence lengths of the polyimide systems.

PI	Sequence Length of the 6FDA/TFDB Segment A	Sequence Length of the 6FDA/SiDA Segment B
A_20_	20	0
(A_2_B_2_)_5_	2	2
(A_5_B_5_)_2_	5	5
A_5_B_10_A_5_	5	10
B_5_A_10_B_5_	10	5
B_20_	0	20

## Data Availability

The data supporting the findings of this study are available within the article and its Supplementary Materials.

## Supplementary Materials

The following supporting information can be downloaded at https://www.mdpi.com/article/10.3390/ijms27167248/s1.
