# Supplementary material for: The Effects of Sequence Structure on the Mechanical Properties of Siloxane-Containing Polyimides: Insights from Molecular Dynamics Simulations
Source: Int J Mol Sci. 2026 Aug 14;27(16):7248. doi: 10.3390/ijms27167248 (PMC13513029; doi:10.3390/ijms27167248)
Supplement: Supplementary file 1 [file ijms-27-07248-s001.zip › ijms-4451356_Supporting_Information_corrected.pdf]

SUPPORTING INFORMATION for

The Effects of Sequence Structure on the Mechanical Properties of Siloxane-containing Polyimides: Insights from Molecular Dynamics Simulations

Lixin Liu [1,2], Song Mo [1], Fan Jia [1,2], Yi Liu [1], Lei Zhai [1] and Lin Fan [1,2,*]

1 Key Laboratory of Science and Technology on High-tech Polymer Materials, Institute of Chemistry, Chinese Academy of Sciences, Beijing 100190, China

2 School of Chemical Sciences, University of Chinese Academy of Sciences, Beijing 100049, China

* Correspondence: fanlin@iccas.ac.cn

Figure S1. Evolution curves of energy for polyimide models during equilibration.

Figure S2. Evolution curves of temperature for polyimide models during equilibration.

Figure S3. Evolution curves of density (a) and radius of gyration (b) for polyimide models during equilibration.

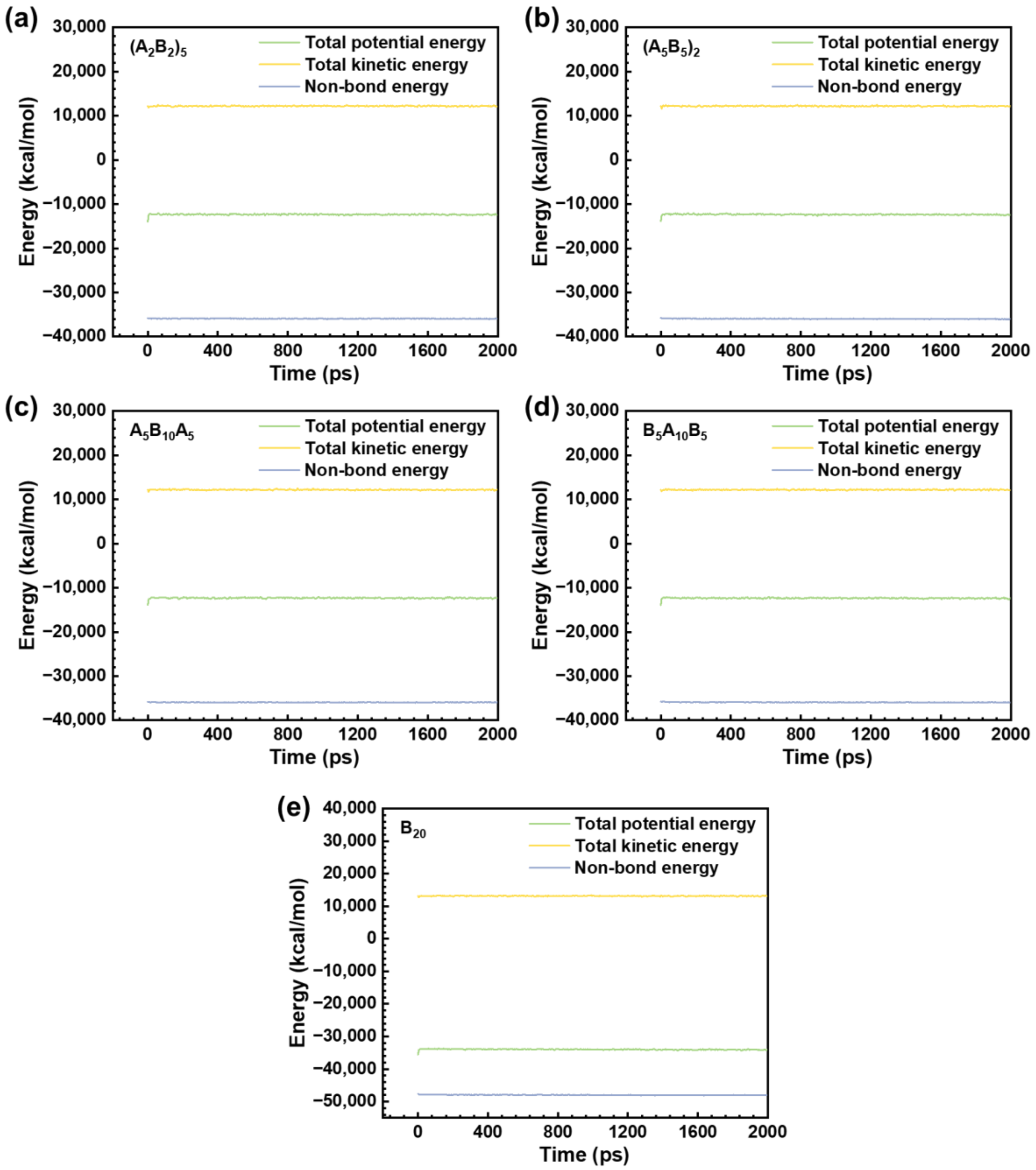

Figure S1. Evolution curves of energy for polyimide models during equilibration.

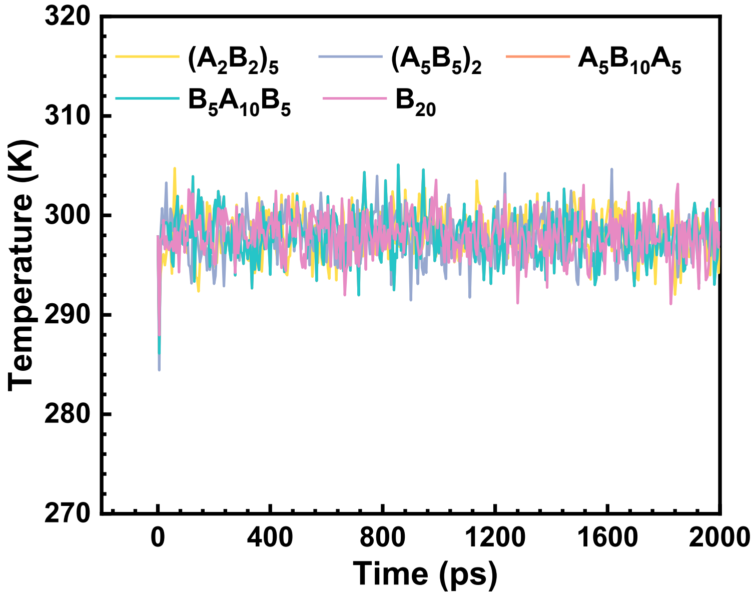

Figure S2. Evolution curves of temperature for polyimide models during equilibration.

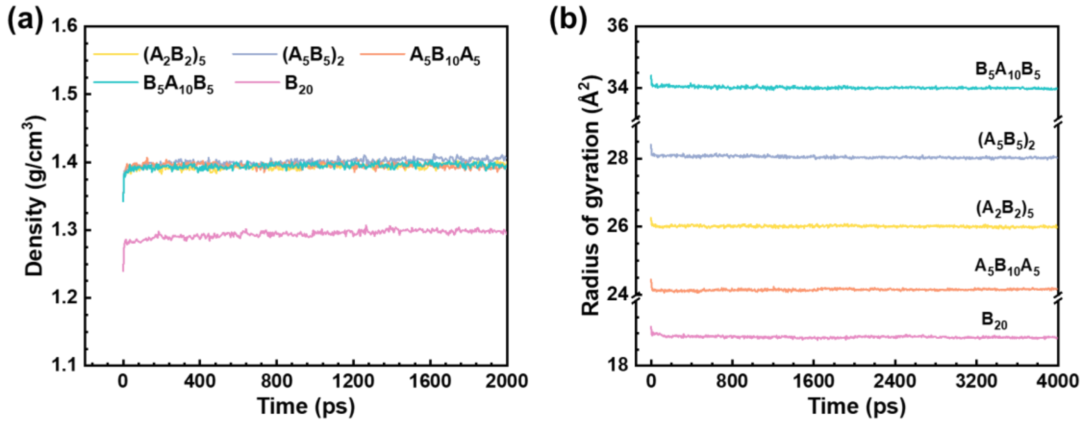

Figure S3. Evolution curves of density (a) and radius of gyration (b) for polyimide models during equilibration.
